# Substituent and Solvent Polarity on the Spectroscopic Properties in Azo Derivatives of 2-Hydroxynaphthalene and Their Difluoroboranes Complexes

**DOI:** 10.3390/ma14123387

**Published:** 2021-06-18

**Authors:** Agnieszka Skotnicka, Przemysław Czeleń

**Affiliations:** 1 Faculty of Chemical Technology and Engineering, UTP University of Science and Technology, Seminaryjna 3, 85-326 Bydgoszcz, Poland; 2 Department of Physical Chemistry, Faculty of Pharmacy, Collegium Medicum, N. Copernicus University, Kurpińskiego 5, 85-950 Bydgoszcz, Poland; przemekcz@cm.umk.pl

**Keywords:** difluoroboranes, substituent effect, synthesis, spectroscopic properties

## Abstract

Novel fluorescent dyes such as difluoroborane complexes of 1-phenylazonaphthalen-2-ol derivatives were successfully synthesized and characterized with a focus on the influence of a substituent and a solvent on the basic photophysical properties. ^1^H, ^11^B, ^13^C, ^15^N, and ^19^F nuclear magnetic resonance (NMR) spectra of substituted 1-phenylazonaphthalen-2-ol difluoroboranes and their parent azo dyes were recorded and discussed. The absorption and emission properties of synthesized compounds were investigated in solvents of varying polarity. They were found to be fluorescent despite the presence of the azo group. The azo group rotation was blocked by complexing with -BF_2_ to get a red shift in absorption. Solvent-dependent spectral properties of compounds were investigated using Lipper-Mataga and Bakhshiev plot. The calculated DFT energies and Frontier Molecular Orbitals calculations of the studied compounds were proved to be consistent with the experimental observations.

## 1. Introduction

Azobenzene with two phenyl rings linked by N=N double bond serves as the parent molecule for the abroad class of aromatic azo compounds. Azo compounds are attractive targets for organic synthesis methodology due to their widespread applications in many areas of technology and medicine. Azo dyes are the most important group of all synthetic dyes that are used extensively for textile dyeing [1,2], as pharmaceuticals [3,4,5,6,7] in organic synthesis [8,9]. Moreover, azobenzenes recently have been targeted for potential applications in areas of nonlinear optics [10], optical storage media [11], chemosensory [12,13], liquid crystals [14], photochemical molecular switches [15,16], and nanotubes [17].

**Scheme 1:**
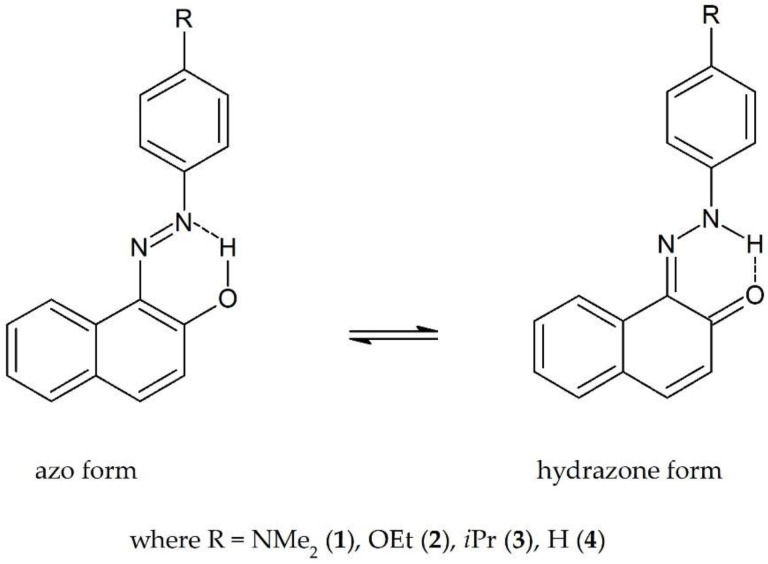
Tautomeric equilibria of 1-arylazonaphthalen-2-ols (**1–4**).

Azobenzene derivatives of 2-naphthol are well-known dyes and can coexist in two tautomeric forms, i.e., azo (enol) and hydrazone (keto) (Scheme 1). These compounds show prototropic tautomerism by an intramolecular proton transfer, which occurs from the hydroxyl oxygen to the azo nitrogen in the case through intramolecular O–H^…^N hydrogen bond. This tautomerization is quite interesting from a theoretical standpoint because the two tautomers have different technical properties. In azonaphthols, the equilibrium depends on temperature, the solvent, and the substitution pattern [18,19,20,21,22].

Many azobenzene derivatives are used as photoresponsive molecular switches by taking advantage of their photoisomerization [23,24]. Their photoisomerization features inhibit another important property of chromophores, fluorescence. If azobenzenes could fluoresce, then they would be useful as fluorescent materials applicable to light-emitting devices, fluorescent probes, and molecular detection because of their easy synthesis and high modification capability.

Organoboron complexes are one of the most important types of fluorescent dyes. Boron complexation is a simple and effective strategy to express or enhance fluorescence. For example, although diketone [25], iminoketone [26], and mentioned earlier azo dyes [27,28] generally show no fluorescence, their boron complexes are known to be fluorescent. It is worth mentioning that many luminescent boron complexes, especially, the famous boron dipyrromethene (BODIPY) dyes, show small Stokes shift and rarely show fluorescence in the solid-state. An interesting alternative may be a new family of aggregation-induced emission (AIE)-active monoboron and bisboron complexes based on hydrazone chelates [27,28].

It is known that systematic change of substituent and benzoannulation have a fundamental impact (qualitative and quantitative) on the properties of compounds exhibiting tautomerism [29,30,31,32]. Presumably, the properties of BF_2_-carrying molecules may also be tuned in this way. This is due to the fact that the proton involved in intramolecular hydrogen bonding can be easily replaced by the BF_2_^+^ cation. The proton-to-BF_2_ exchange thus creates an opportunity for some new dyes also in azobenzene derivatives of 2-hydoxynaphthalene.

**Scheme 2:**
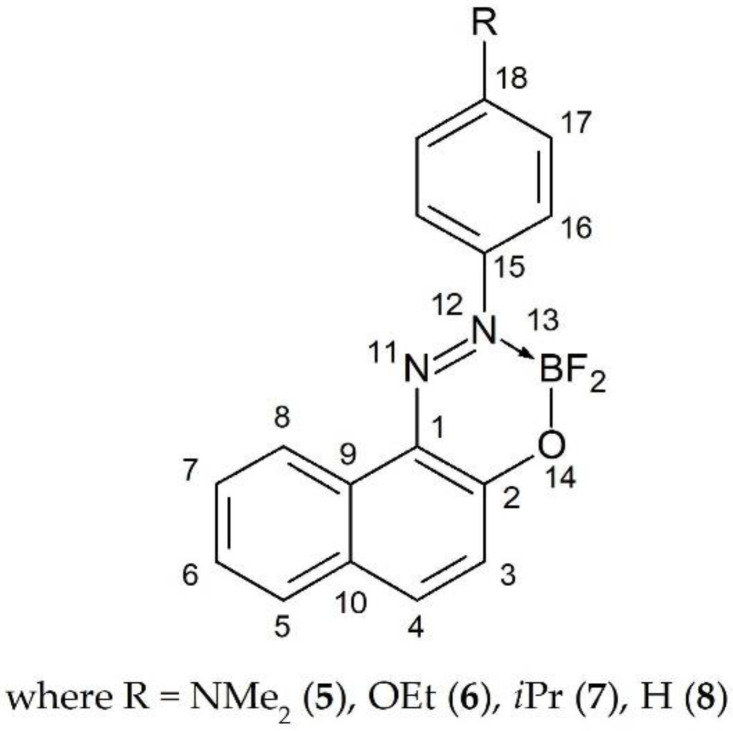
Structure and atom numbering in (**5–8)**.

The main motivation to undertake the current study is to describe the photochemical properties of 1-phenylazonaphthalen-2-ols difluoroboranes (**5–8**) (Scheme 2) in solutions of different polarity and to compare them with non-BF_2_ chelated pattern compounds (**1–4**). In the present work, the synthesis of phenylazonaphthalen-2-ols (**1–4**) and their difluoroboranyl derivatives (**5–8**) was successfully performed. The structures of the newly synthesized dyes were confirmed by the spectroscopic technique of ^1^H, ^11^B, ^13^C, ^15^N, and ^19^F NMR. Additionally, we demonstrate how absorption and emission spectra (position, intensity, and shape) of investigated compounds are changed by the solvents in different polarity. The details about synthesis, characterization, solvatochromic, and photophysical properties of investigated compounds are presented and discussed below.

## 2. Materials and Methods

### 2.1. Materials

All reagents and solvents were purchased from Sigma-Aldrich (Poznań, Poland) and used without further purification. The highest (≥99%) purity of all used chemicals was required for spectroscopic studies.

### 2.2. Synthesis

#### 2.2.1. Synthesis of 1-Arylazonaphthalen-2-ols (**1**–**4**)—General Procedure

The mixture of 1 mmol substituted aniline in 37% HCl (5 mL) was stirred in an ice-bath and then the solution of 1.1 mmol sodium nitrite in water (5 mL) was added slowly (the temperature was not allowed to rise above 5 °C) for the formation of diazonium salt, which was allowed to react with 1 mmol naphthalen-2-ol in 10% NaOH (15 mL) solution. The reaction mixture was stirred at 0–5 °C for 10 min and then under an ambient temperature for 30 min. The reaction progress was monitored by thin-layer chromatography (TLC) using a mixture of EtOAc and *n*-hexane (1:1; V/V) as a solvent. Following the disappearance of the starting materials, the reaction mixture was filtrated and washed with water. The crude solid was recrystallized from ethanol.

#### 2.2.2. Elemental Analysis Is as Follows

1-(4-Dimethylamino)phenylazonaphthalen-2-ol (**1**) Dark brown solid, yield 80%, m.p. 172–173 °C (179–181 °C [33], 180–182 °C [34], 183–184 °C [35]). ^1^H NMR (CDCl_3_ from TMS) δ (ppm): 15.52 (s, 1H), 8.81 (d, 1H, ^3^*J*_H_,_H_ = 8.44 Hz), 7.87 (m, 2H), 7.75 (m, 2H), 7,59 (m, 1H), 7.42 (m, 1H), 7.13 (d, 1H, ^3^*J*_H_,_H_ = 9.04 Hz), 6.72 (s, 2H), 3.13 (s, 6H). ^13^C NMR δ (ppm): 156.5, 151.6, 139.7, 134.2, 133.0, 129.4, 128.2, 128.1, 127.5, 124.2, 123.1, 121.7, 121.1, 112.2, 40.4. ^15^N NMR (CDCl_3_ from MeNO_2_) δ (ppm): −121.88, −392.85 (NMe_2_). C_18_H_17_N_3_O, Calcd. C, 74.20; H, 5.88; N, 14.49. Found C, 74.31; H, 5.83; N, 14.44.

1-(4-Ethoxy)phenylazonaphthalen-2-ol (**2**) Red solid, yield 82%, m.p. 132–134 °C (133–134 °C [36,37]). ^1^H NMR (CDCl_3_ from TMS) δ (ppm): 15.66 (s, 1H), 8.64 (d, 1H, ^3^*J*_H,H_ = 8.40 Hz), 7.75 (m, 2H), 7.69 (d, 1H, ^3^*J*_H,H_ = 9.16 Hz), 7,62 (d, 1H, ^3^*J*_H,H_ = 7.88 Hz), 7.50 (m, 1H), 7.33 (m, 1H), 6.98 (s, 1H), 6.94 (m, 2H), 4.06 (q, 2H), 1.39 (t, 3H). ^13^C NMR δ (ppm): 161.1, 160.1, 141.8, 136.6, 133.3, 129.5, 128.3, 128.2, 128.1, 124.8, 122.1, 121.6, 115.3, 63.9, 14.8. ^15^N NMR (CDCl_3_ from MeNO_2_) δ (ppm): −129.44. C_18_H_16_N_2_O_2_, Calcd. C, 73.95; H, 5.52; N, 9.58. Found C, 73.82; H, 5.63; N, 9.60.

1-(4-Isopropylo)phenylazonaphthalen-2-ol (**3**) Red solid, yield 83%, m.p. 69–70 °C. ^1^H NMR (CDCl_3_ from TMS) δ (ppm): 16.24 (s, 1H), 8.64 (d, 1H, ^3^*J*_H,H_ = 8.28 Hz), 7.74 (m, 3H), 7.66 (d, 1H, ^3^*J*_H,H_ = 7.76 Hz), 7.59 (m, 1H), 7.42 (m, 1H), 7.37 (m, 2H), 6.95 (d, 1H, ^3^*J*_H,H_ = 9.36 Hz), 3.00 (m, 1H), 1.32 (d, 6H, ^3^*J*_H,H_ = 6.95 Hz). ^13^C NMR δ (ppm): 168.5, 149.3, 143.8, 138.9, 133.6, 129.8, 128.6, 128.5, 128.1, 127.6, 125.33, 124.0, 121.6, 119.3, 33.9, 23.9. ^15^N NMR (CDCl_3_ from MeNO_2_) δ (ppm): −117.27. C_19_H_18_N_2_O, Calcd. C, 8.59; H, 6.25; N, 9.65. Found C, 8.74; H, 6.10; N, 9.65.

1-Phenylazonaphthalen-2-ol (**4**) Red solid, yield 88%, m.p. 131–132 °C (130–132 °C [38], 130–131 °C [39]). ^1^H NMR (CDCl_3_ from TMS) δ (ppm): 16.30 (s, 1H), 8.59 (d, 1H, ^3^*J*_H_,_H_ = 8.59 Hz), 7.76 (m, 3H), 7.63 (d, 1H, ^3^*J*_H_,_H_ = 7.76 Hz), 7.58 (m, 1H), 7.51 (m, 2H), 7.42 (m, 1H), 7.33 (m, 1H), 6.89 (d, 1H, ^3^*J*_H_,_H_ = 9,44 Hz). ^13^C NMR δ (ppm): 171.8, 144.8, 140.0, 133.6, 130.1, 129.6, 128.9, 128.6, 128.1, 127.4, 125.7, 124.8, 121.7, 118.6. ^15^N NMR (CDCl_3_ from MeNO_2_) δ (ppm): −120.36. C_16_H_12_N_2_O, Calcd. C, 77.40; H, 4.87; N, 11.28. Found C, 77.36; H, 4,95; N, 11,24.

#### 2.2.3. Synthesis of 1-Phenylazonaphthalen-2-ols Difluoroboranes (**5**–**8**)—General Procedure

The typical procedure was as follows: BF_3_ etherate (five equivalents) was added to the magnetically stirred solution (nitrogen atmosphere) of substituted 1-phenylazonaphthalen-2-ol (1 g) in dry dichloromethane (15–20 mL) and *N*,*N*-diisopropylethylamine (five equivalents). Then the solution was stirred overnight at room temperature and concentrated Na_2_CO_3_ water solution (20 mL) was added slowly to the mixture. The organic fraction was separated, the water layer extracted with chloroform (two times using ca. 20–30 mL), dried (with Na_2_SO_4_), and evaporated under reduced pressure. The residual solids were washed by using a mixture of *n*-hexane and EtOAc (5:1; V/V) solution twice and purified by flash chromatography (SiO_2_) using dichloromethane (DCM) as the eluent.

#### 2.2.4. Elemental Analysis Is as Follows

1-(4-Dimethylamino)phenylazonaphthalen-2-ole Difluoroborane (**5**) Dark purple solid, yield 38%, m.p. 207–209 °C. ^1^H NMR (CDCl_3_ from TMS) δ (ppm): 8.59 (d, 1H, ^3^*J*_H_,_H_ = 8.36 Hz), 8.06 (d, 2H, ^3^*J*_H_,_H_ = 9.36 Hz), 7.93 (d, 1H, ^3^*J*_H_,_H_ = 9.00 Hz), 7.73 (d, 1H, ^3^*J*_H_,_H_ = 8.04 Hz), 7.60 (m, 1H), 7.44 (m, 1H), 7.17 (d, 1H, ^3^*J*_H_,_H_ = 9.04 Hz), 6.71 (m, 2H), 3.07 (s, 6H). ^11^B NMR (CDCl_3_ from BF_3_·Et_2_O) δ (ppm): −0.12 (t). ^13^C NMR δ (ppm): 152.1, 149.8, 139.6, 135.6, 131.7, 130.2, 129.2, 128.9, 128.7, 125.7, 125.1, 121.4, 119.7, 112.0, 40.3. ^19^F NMR (CDCl_3_ from CFCl_3_) δ (ppm): −134.05. ^15^N NMR (CDCl_3_ from MeNO_2_) δ (ppm): −90.10, −315.69 (NMe_2_). C_18_H_16_BF_2_N_3_O, Calcd. C, 63.75; H, 4.76; N, 12.39. Found C, 63.88; H, 4.50; N, 12.52.

1-(4-Ethoxy)phenylazonaphthalen-2-ole Difluoroborane (**6**) Red solid, yield 29%, m.p. 139–140 °C. ^1^H NMR (CDCl_3_ from TMS) δ (ppm): 8.58 (d, 1H, ^3^*J*_H_,_H_ = 8.36 Hz), 8.04 (m, 3H), 7.76 (d, 1H, ^3^*J*_H_,_H_ = 8.00 Hz), 7.65 (m, 1H), 7.48 (m, 1H), 7.18 (m, 2H), 6.95 (m, 2H), 4.08 (q, 2H), 1.42 (t, 3H). ^11^B NMR (CDCl_3_ from BF_3_·Et_2_O) δ (ppm): −0.10 (t). ^13^C NMR δ (ppm): 161.5, 151.5, 142.3, 138.8, 131.9, 130.5, 130.0, 129.0, 128.8, 126.3, 124.9, 121.3, 119.7, 115.1, 64.1, 14.7. ^19^F NMR (CDCl_3_ from CFCl_3_) δ (ppm): −93.11. ^15^N NMR (CDCl_3_ from MeNO_2_) δ (ppm): −93.11. C_18_H_15_BF_2_N_2_O_2_, Calcd. C, 63.19; H, 5.01; N, 8.19. Found C, 63.28; H, 5.08; N, 8.03.

1-(4-Isopropylo)phenylazonaphthalen-2-ole Difluoroborane (**7**) Red solid, yield 21%, m.p. 115–117 °C. ^1^H NMR (CDCl_3_ from TMS) δ (ppm): 8.57 (d, 1H, ^3^*J*_H_,_H_ = 8.28 Hz), 8.06 (d, 1H, ^3^*J*_H_,_H_ = 9.08 Hz), 7.97 (d, 2H, ^3^*J*_H_,_H_ = 8.56 Hz), 7.53 (d, 1H, ^3^*J*_H_,_H_ = 8.00 Hz), 7.65 (m, 1H), 7.49 (m, 1H), 7.33 (d, 2H, ^3^*J*_H_,_H_ = 8.64 Hz), 7.18 (m, 1H) 2.94 (m, 1H), 1.24 (d, 6H, ^3^*J*_H,H_ = 6.92 Hz). ^11^B NMR (CDCl_3_ from BF_3_·Et_2_O) δ (ppm): −0.06 (t). ^13^C NMR δ (ppm): 151.6, 151.4, 142.2, 131.0, 129.2, 128.1, 127.8, 126.6, 126.5, 125.4, 122.1, 120.4, 118.8, 33.1, 22.9. ^19^F NMR (CDCl_3_ from CFCl_3_) δ (ppm): −132.33. ^15^N NMR (CDCl_3_ from MeNO_2_) δ (ppm): −95.23. C_19_H_17_BF_2_N_2_O, Calcd. C, 67.08; H, 5.63; N, 8.24. Found C, 67.00; H, 5.55; N, 8.40.

1-Phenylazonaphthalen-2-ole Difluoroborane (**8**) Red solid, yield 40%, m.p. 143–145 °C (175–176 °C [40,41,42]). ^1^H NMR (CDCl_3_ from TMS) δ (ppm): 8.63 (d, 1H, ^3^*J*_H_,_H_ = 7.84 Hz), 8.19 (d, 1H, ^3^*J*_H_,_H_ = 9.08 Hz), 8.14 (m, 2H), 7.87 (d, 1H, ^3^*J*_H_,_H_ = 8.00 Hz), 7.77 (m, 1H), 7.59 (m, 4H), 7.31 (s, 1H). ^11^B NMR (CDCl_3_ from BF_3_·Et_2_O) δ (ppm): −0.11 (t). ^13^C NMR δ (ppm): 152.9, 145.2, 143.9, 132.1, 131.2, 130.9, 130.4, 129.4, 129.2, 128.8, 126.6, 123.1, 121.4, 119.8. ^19^F NMR (CDCl_3_ from CFCl_3_) δ (ppm): −131.94. ^15^N NMR (CDCl_3_ from MeNO_2_) δ (ppm): −95.29. C_16_H_11_BF_2_N_2_O, Calcd. C, 64.91; H, 3.74; N, 9.46. Found C, 65.07; H, 3.84; N, 9.20.

### 2.3. Measurements

The ^1^H-NMR spectra were recorded using an Ascend III spectrometer operating at 400 MHz, Bruker (Bydgoszcz, Poland). Chloroform was used as a solvent and tetramethylsilane (TMS) as the internal standard. Chemical shifts (δ) were reported in ppm relative to TMS and coupling constants (J) in Hz.

The elemental analysis was made with a Vario MACRO 11.45–0000, Elemental Analyser System GmbH (Toruń, Poland), operating with the VARIOEL software (version 5.14.4.22) (Toruń, Poland)

The melting point was measured on the Melting Point M-565 Apparatus (Buchi) (Bydgoszcz, Poland) with a measuring speed of 5 °C/min.

The absorption and emission spectra were measured at room temperature in a quartz cuvette (1 cm) using an Agilent Technology UV–Vis Cary 60 Spectrophotometer (Bydgoszcz, Poland) and a Hitachi F-7000 Spectrofluorometer (Bydgoszcz, Poland), respectively.

The fluorescence quantum yields for the compounds in chloroform were determined as follows, the fluorescence spectrum of diluted (A ≈ 0.1) dyes solution was recorded by excitation at the absorption band maximum of the reference. Diluted rhodamine 6G in ethanol (*ϕ* = 0.88) [43] was used as a reference. The fluorescence spectrum of rhodamine 6G was obtained by excitation at its absorption peak at 488 nm. The quantum yield of the tested compounds (*ϕ_dye_*) was calculated using the following equation [44]:

(1) $$\phi_{dye}=\phi_{ref}\;.\;\frac{I_{dye}}{I_{ref}}\frac{A_{ref}}{A_{dye}}\;.\;\frac{n_{dye}^{\;\;\;\;2}}{n_{ref}^{\;\;\;\;2}}$$

where *ϕ_ref_* is the fluorescence quantum yield of the reference sample rhodamine 6G in ethanol, *A_dye_* and *A_ref_* are the absorbance of the dye and reference samples at the excitation wavelengths (355 nm), *I_dye_* and *I_ref_* are the integrated emission intensity for the dyes and references sample, *n_dye_* and *n_ref_* are the refractive indices of the solvents used for the dyes and reference, respectively. Cresyl violet in ethanol (*ϕ* = 0.51 [43] *λ_ex_* = 578 nm) and coumarin I in ethanol (*ϕ* = 0.64 [43] *λ_ex_* = 404 nm) were used as a reference standard for 1-(4-dimethylamino)phenylazonaphthalen-2-ole difluoroborane (**5**) and 1-(4-ethoxy)phenylazonaphthalen-2-ol (**2**), respectively.

The brightness was calculated using the following equation [45]:

(2) Brightness= ɛ · ϕ

where *ϕ* is the fluorescence quantum yield and *ɛ* is the molar extinction coefficient.

The experimental excited-state dipole moments of **1–8** in solvents of varying polarity were determined according to Lippert’s and Bakhshiev’s equations. Lippert’s equation [46,47] can be expressed as:

(3) $$∆v=\frac{2{\left(\mu_{e}-\mu_{g}\right)}^{2}}{hca^{3}}\;\left(\frac{\varepsilon -1}{2\varepsilon +1}-\frac{n^{2}-1}{2n^{2}+1}\right)$$

In which *∆v* = *v_abs_*−*v_em_* stands for Stokes shift, *v_abs_* and *v_em_* are the absorption and emission frequency (cm^−1^), *μ_g_* and *μ_e_* refer to the dipole moments of the ground- and excited states, respectively, *h* is the Planck’s constant, *c* is the velocity of light in vacuum, *a* is the Onsager radius, *ɛ* is the refractive index, and *n* is the dielectric constant.

Bakhshiev’s equation [47] reads as:

(4) $$∆v=\frac{2{\left(\mu_{e}-\mu_{g}\right)}^{2}}{hca^{3}}\;\left[\frac{\varepsilon -1}{\varepsilon +2}-\frac{n^{2}-1}{n^{2}+2}\right]\;\frac{\left(2n^{2}+1\right)}{\left(n^{2}+2\right)}$$

### 2.4. Quantum-Mechanical Calculations

The geometry optimization and the Highest Occupied Molecular Orbital (HOMO) and Lowest Unoccupied Molecular Orbital (LUMO) energies were calculated based on density functional theory with the use of B3LYP [48,49,50] functional and the 6–311++G(d,p) basis set [51,52]. The calculations including solvation were realized with the use of a SCRF (self-consistent reaction field) approach [53] based on the Poisson–Boltzmann equation [54]. All calculations were realized with the use of Gaussian 09 software [55]. The Avogadro 1.2.0 application [56] was used during the analysis of the frontier orbitals.

## 3. Results and Discussion

### 3.1. Synthesis and Identification of 1-Phenylazonaphthalen-2-ols (**1**–**4**) and Their Difluoroboranes (**5**–**8**)

**Scheme 3:**
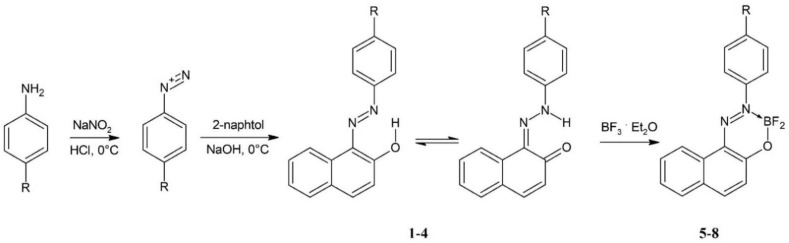
The schematic representation of the synthesis of difluoroboranes substituted 1-phenylazonaphthalen-2-ols. (**1–8**), where R = NMe_2_ (**1**,**5**), OEt (**2**,**6**), *i*Pr (**3**,**7**), H (**4**,**8**).

In recent years, several methods have been reported for the synthesis of aromatic azo components [8,57,58,59,60]. Although remarkable developments have been achieved in this field still the most important method for the preparation of these classes of compounds is the diazotization-azo coupling reaction. In the presented paper, azo compounds were synthesized by coupling 2-naphthol with the corresponding diazonium salt and recrystallization in ethanol. The tautomeric mixture of 1-phenylazonaphthalen-2-ols (**1–4**) was then allowed to react with the boron trifluoride diethyl ether complex in the presence of *N*,*N*-diisopropylethylamine (DIPEA) in dry dichloromethane to afford the corresponding BF_2_ complexes (**5–8**). The general route for the synthesis of NBF_2_O derivatives is presented in Scheme 3.

The structures of (**1–8**) were confirmed by their ^1^H, ^13^C, and ^15^N NMR spectral analyses. Additionally, ^11^B and ^19^F NMR spectra were recorded for complexes (**5–8**) (see details in the Materials and Methods section). As previously reported [18,19,20,21,22], the compounds showed in Scheme 1 were present in solution as a proton transfer equilibrium. In all cases, their ^1^H NMR spectra showed a signal with chemical shift in the range of 15.66–16.62 ppm, corresponding to the O–H^...^N proton involved in the relevant intramolecular hydrogen bond. The ^11^B NMR signals showing near −0.10 ppm and quadruplet at around −132 ppm were observed in ^19^F NMR of each difluoroborane complex, confirm replacing the O–H^...^N proton in structures (**5–8**) by the BF_2_^+^ cation.

### 3.2. Spectroscopic Properties

**Figure 1:**
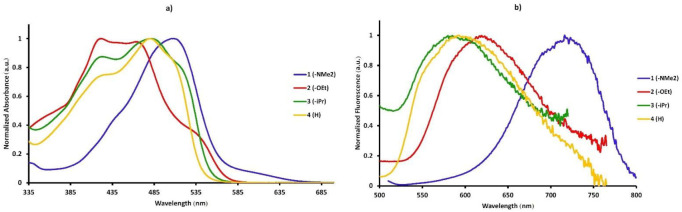
The normalized UV–Vis absorption of main bands (**a**) and fluorescence (**b**) spectra of **1–4** in DMSO.

**Figure 2:**
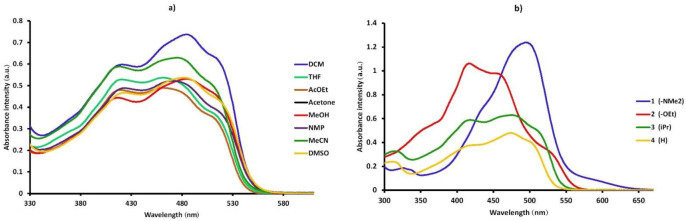
Absorption intensity spectra of 1-(4-isopropylo)phenylazonaphthalen-2-ol (**3**) in solvents of different polarity. (**a**) and absorption intensity spectra of **1–4** in acetonitrile (**b**) (5 × 10^−5^ M).

**Figure 3:**
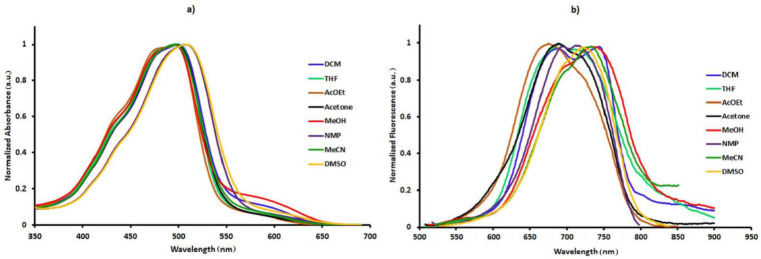
Normalized absorption (**a**) and fluorescence (**b**) spectra of 1-(4-dimethylamino)phenylazonaphthalen-2-ol (**1**) in solvents of different polarity.

The compounds were studied for their photophysical properties in solvents of various polarity and nature. The absorption properties of compounds **1–4** were summarized in Table 1. Generally, the electronic absorption spectra of these compounds in solution exhibit two types of bands. The shorter wavelength band in the UV region of 247–326 nm was ascribed mainly to the π–π* transition of the aromatic system present in the structure of the studied dyes. The second band observed in the region of 335–635 nm (Figure 1a) was assigned to n–π* transition with a considerable charge-transfer character (CT transition) [61]. The charge-transfer nature of this band was deduced from its broadness, as from the sensitivity of its *λ_max_* to the type of substituent attached to the azo coupler [62]. Based on the results presented in Table 1, the solvent polarity only weakly affected the position of the maximum absorption band. The increase in solvent polarity caused a slight bathochromic shift of the long-wave absorption band. The highest absorption intensity of azo dyes **1–4** was observed in dichloromethane, acetonitrile and tetrahydrofuran (Figure 2a). As expected, compound **1** has the biggest redshift (at around 500 nm) in all solvents as compared to the rest compounds (Figure 3a). It was previously indicated that the presence of both electron-withdrawing and electron-donating groups caused a redshift in these compounds [46]. It was accompanied by an increase in the absorption intensity (Figure 2b). The molar extinction the dyes **1–4** did not achieve relatively high values. This parameter ranged from 0.76 × 10^4^ to 2.85 × 10^4^ M^−1^ cm^−1^ (Table 1). It did not show any clear correlation between the solvent polarities and the molar extinction coefficients.

**Figure 4:**
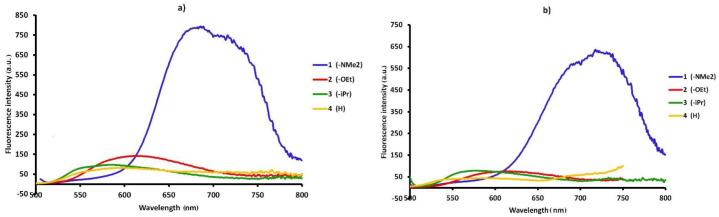
Fluorescence intensity spectra of **1–4** in dichloromethane (**a**) and in acetonitrile (**b**) (5 × 10^−5^ M).

The emission wavelengths of the dyes **1–4**, similar to absorption wavelengths, were not solvent dependent. The emissions of all described compounds ranged between 540 and 800 nm (Figure 1b). The changes in Stokes shifts related to the nature of the substituent in phenyl ring were also reflected in changes in the fluorescence quantum yields (Scheme 1). The unsubstituted compound **4** (R = H), e.g., in tetrahydrofuran had a fluorescence quantum yield of 1.01 × 10^−4^ and exhibited a Stokes’ shift of 4163 cm^−1^. Introducing an electron-donating substituent enhanced the fluorescence quantum yields and Stokes’ shift, e.g., for –NMe_2_ (**1**) the *ϕ_fl_* = 0.0084 and Stokes’ shift equal to 5547 cm^−1^ (in THF), respectively. The same dependence of the substituent effect on the fluorescence intensity (Figure 4) as on the absorption intensity was observed for compounds **1–4**.

**Figure 5:**
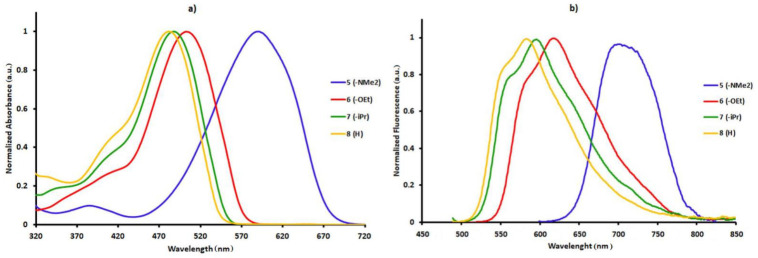
The normalized UV–Vis absorption of main bands (**a**) and fluorescence (**b**) spectra of **5–8** in DCM.

**Figure 6:**
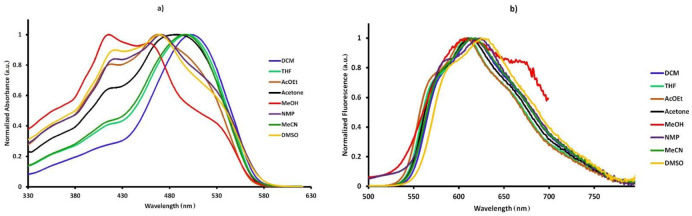
Normalized absorption (**a**) and fluorescence (**b**) spectra of 1-(4-ethoxy)phenylazonaphthalen-2-ole difluoroborane (**6**) in solvents of different polarity.

**Figure 7:**
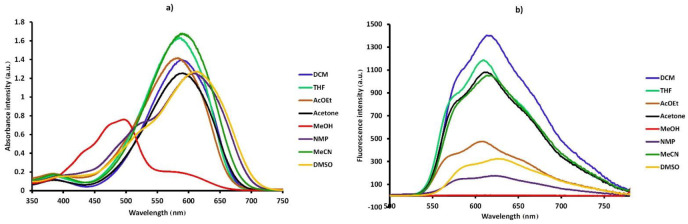
Absorption (**a**) and fluorescence (**b**) intensity spectra of 1-(4-ethoxy)phenylazonaphthalen-2-ole difluoroborane (**6**) in solvents of different polarity.

The dyes **5–8** are the BF_2_-complex derivatives of the dyes **1–4** absorbed at a longer wavelength. The differences between the two kinds of chromophores are the rigidization of the azo group and the presence of an electron-deficient BF_2_-core, which formed a more efficient acceptor [46] in the case of dyes **5–8**. The biggest bathochromic shift was observed for dyes **5** and **6** (with the most electron-donating substituent) [42]. They absorbed at about 60–100 nm longer wavelengths compared to their parent, non-complexed dyes (Figure 5a). In this case, the maximum of absorbance shifted towards higher wavelength values as the polarity of solvent increased (Figure 6a). The fluorescence emission of **5–8** exceeded 550 nm in all the solvents tested. The emission spectra (Figure 5b) were broad with the single maximum of fluorescence (*λ_fl_*) at about 570–610 nm for **6–8** and at about 690–760 nm for **5**. The polarity of the solvent did not significantly influence the position of the fluorescence band (Figure 6b). The highest absorption and fluorescence intensity of **5–8** were observed in dichloromethane, acetonitrile, and tetrahydrofuran (Figure 7). The fluorescence quantum yield has drastically improved in azo-BF_2_-complexes over the azo compounds, the order of the increase in quantum yield was about 10–100 folds. The quantum yields were the highest in the –NH_2_ and −OEt substituted derivatives (**5** and **6**) in their respective series and all solvents. The calculated brightness confirmed the trend shown. Table 2 collected the values of the absorption maximum positions (*λ_ab_*), the fluorescence maximum positions (*λ_fl_*), Stokes shifts, the molar extinction coefficient (*ɛ*), the fluorescence quantum yields (*ϕ_fl_*), and the brightness of substituted difluoroboranes of 1-phenylazonaphthalen-2-ols (**5–8**).

### 3.3. Solvatochromism

The solvatochromic behavior of the dyes **1–4** and **5–8** was studied with the help of Lippert [46,63] and Bakhshiev [63] plots. Lippert’s theory assumes that general solvent effects are present in the solvent medium and the polarizability of the solute molecule is neglected. This model does not contain any chemical interactions. The direction of ground- and excited-dipole moments is parallel to each other, namely, it is collinear. Deviations from Lippert’s theory occur due to specific solute–solvent interactions such as hydrogen bonding or a formation of charge-transfer states [47,64].

Bakhshiev’s theory takes into account the solute polarizability besides specific solute–solvent interactions [64]. The direction of dipole moments of ground- and excited-dipole state is not collinear, but the linearity of the dipoles is close to each other. Deviations from the linearity of Bakhshiev’s equation may result from an incomplete solvent relaxation before to fluorescence emission or specific solute–solvent interactions, such as hydrogen bonding [65].

Lippert-Mataga and Bakhshiev plots showed nonlinear nature of the plot, which was observed in *N*-methyl-2-pyrrolidone for all compounds **1–8**, while such behavior for BF_2_-complexes **5–8** was observed additionally in methanol. Deviations from linearity they occurred especially in the presence of polar solvents. These deviations were related to the extent of interactions between the solute and solvent molecules, in this case the formation of hydrogen bonds between them. Only compound **3** exhibited similar nature to that of **4** (Lippert-Mataga and Bakhshiev plots of compounds **1–8** in different solvents are available in Supplementary Materials).

Lippert-Mataga and Bakhshiev plots showed unsatisfactory linearity of Stokes shift vs. *f_1_* (Lippert plots) and *f_2_* (Bakhshiev plots), respectively functions with low regression coefficients (R^2^ ≤ 0.5654) suggested a slight effect of solvent polarity on Stokes shift. Stokes shift decreased for **3** and **8**, while increased for **1**, **5,** and **6** with increasing solvent polarity (Figures S19 and S20 in Supplementary Materials).

It is worth noting that the BF_2_ complexes of azo dyes, i.e., **5–8** showed no correlation with the polarity of the solvent. Plots of the Stokes shift in cm^−1^ versus the solvent polarity parameter *f_1_* (Lippert plots) and *f_2_* (Bakhshiev plots) were with very low regression coefficients. This implied that in the boron complexes due to rigidity the emissive state was not an ICT state but a local relaxed excited state. The lower (as compared to compound **1–4**) Stokes shift exhibited by the dyes in all the solvents added to the justification [46].

### 3.4. Computational Details

The 1-phenylazonaphthalen-2-ols considered (**1–4**) in this work can coexist in two tautomeric forms. The energetic characteristics of azo and hydrazone forms estimated during optimization in DMSO solvent were presented in Table 3. All obtained energies unambiguously showed that hydrazone forms were characterized by lower energy values and were more stable than corresponding azo structures. The analysis of all values indicated that the presence of chemical groups with higher electron-donating character caused a decrease of energy differences between compared tautomeric forms. This supports previous reports [18,19,20,21,22].

**Figure 8:**
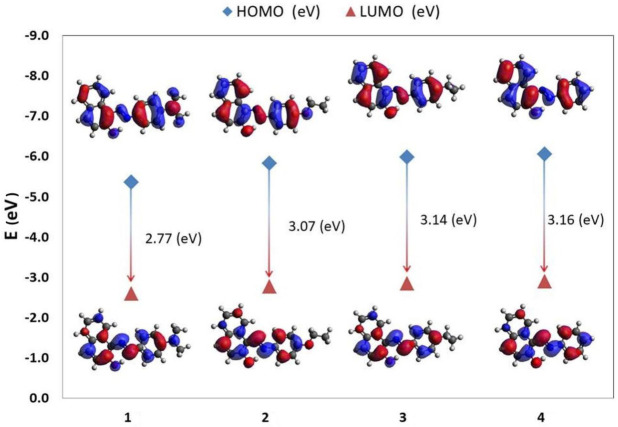
Graphic representation of HOMO and LUMO orbitals for azo forms of 1-phenylazonaphthalen-2-ols (**1–4**) calculated in DMSO.

**Figure 9:**
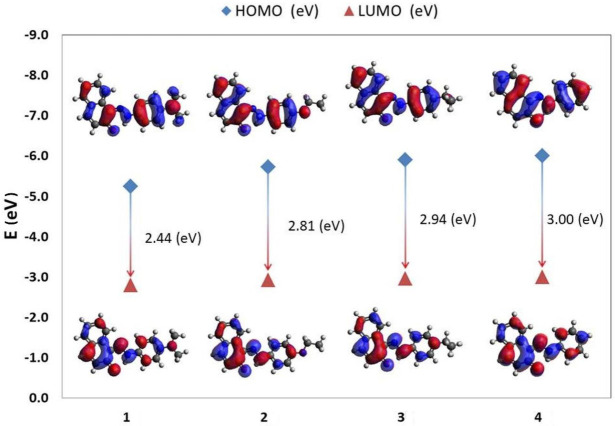
Graphic representation of HOMO and LUMO orbitals for hydrazone forms of 1-phenylazonaphthalen-2-ols (**1–4**) calculated in DMSO.

**Figure 10:**
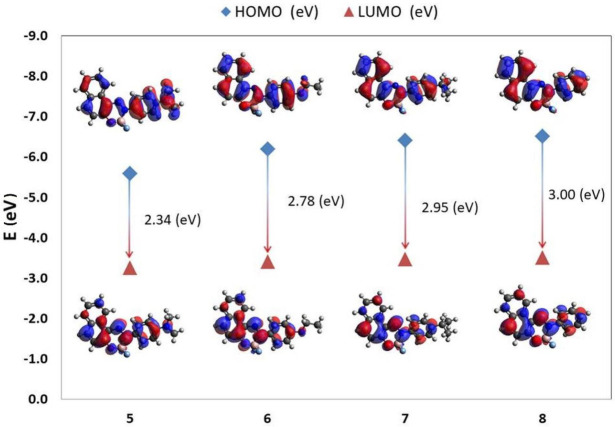
Graphic representation of HOMO and LUMO orbitals for difluoroboranes of 1-phenylazonaphthalen-2-ols (**5–8**) calculated in DMSO.

The characteristics of frontier molecular orbital properties including energy levels of HOMO, LUMO, and other descriptors [66] gave insight into spectroscopic properties of considered dyes. Complex presentation of such data was presented in Figure 8, Figure 9 and Figure 10 respectively, and in Table 4. Based on the accumulated values, for all considered sets of molecules, including both tautomeric forms of native dyes and their difluoroborane derivatives, analogous relation between the presence of electron-donating groups and increase of energy levels of frontal orbitals (NMe_2_ > OEt > *i*Pr > H) was observed. The observed increase in energy for the HOMO and LUMO orbitals is accompanied by a simultaneous reduction in the difference in energy levels between the mentioned types of orbitals, which translated into a simultaneous decrease of energy gap and hardness values. The observed relationship was intensified for the considered groups of molecules in the following order azo < hydrazono < difluoroborane derivatives. The presence of substituents with strong electron-donating character (–NMe_2_; –OEt) was the source of strong bathochromic shift, which confirmed the decrease of energy gap values of substituted dyes relative to the native molecules (ΔE gap _(**5–8)**_ = 0.667 eV; ΔE gap _(**6–8**)_ = 0.220 eV). The computational data well correlate with outcomes obtained during the experimental stage, which are presented in Figure 5 and Table 2. Analysis of the absorbance maximum values clearly confirms dependencies identified during the computational stage (*λ_ab_*_**5** (–NMe2)_ = 591 nm; *λ_ab_*_**6** (–OEt)_ = 502 nm; *λ_ab_*_**8** (–H)_ = 482 nm). Calculations taking into account solvents with extreme polarity values, namely DMSO and DCM, allowed us to assess their influence on spectroscopic properties of considered dyes (Table 4). In order to verify the influence of hydrogen bonds in the solvation environment on the spectroscopic properties of the analyzed compounds, calculations were also performed for methanol.

Obtained values showed that dyes with –NMe_2_ (**1** and **5**) active group exhibited noticeable changes of energy gap values indicating the correlation of increase of solvent polarity with positive solvatochromism of considered molecules (**5** from Eg _(DCM)_ = 2.366 eV to Eg _(DMSO)_ = 2.336 eV). The confirmation of these observations lies in experimental values of absorbance maximum for the analyzed dye *λ_ab_*_DMSO_ = 610 nm and *λ_ab_*_DCM_ = 591 nm (Compound **5** Table 2). Similar relationships, but of much lower intensity, were observed for the other dyes.

## 4. Conclusions

Four novel difluoroborane complexes dyes (**5–8**) based on 1-phenylazonaphthalen-2-ol derivatives were successfully synthesized. Complexes were identified using a magnetic atomic nucleus ^1^H, ^11^B, ^13^C, ^15^N, and ^19^F isotope resonance spectra. The BF_2_-complexes **5–8** exhibited red-shifted absorption maxima from 458 to 610 nm and a slightly higher molar extinction coefficient as compared to parent dyes **1–4**, respectively. All of 1-phenylazonaphthalen-2-ols difluoroboranes emitted in the far-red region (550–760 nm) whereas only **1** weakly emitted at 673–727 nm. Among all derivatives, only 1-(4-dimethylamino)phenylazonaphthalen-2-ole difluoroborane **5** (the NMe_2_ group was the strongest electron donor in the series) demonstrated relatively high fluorescence. Dye **3** and **8** showed negative solvatochromism, while dyes **1**, **5**, and **6** showed positive solvatochromism. It was shown that the BF_2_-complexes (**5–8**), which are the rigidized versions of the azo dyes (**1–4**), did not show any linear relations with the solvent polarity parameters based on dielectric constant and refractive index. Transformation of azo compounds with electron-withdrawing substituents (R = Br, F, and NO_2_) into their BF_2_ complexes turned out to be impossible. The calculated DFT energies and the frontier molecular orbital calculations of the studied compounds showed to be consistent with the experimental observations and confirmed the insignificant influence of the polarity of the solvents on the spectroscopic properties.

## Figures and Tables

**Table 1:** Photophysical properties of compounds **1**–**4**.

No.	Substituent	Solvent	*λ_ab_* (nm)	*λ_fl_* (nm)	*ɛ* (× 10^4^, M^−1^·cm^−1^)	Stokes Shift (cm^−1^)	*ϕ_fl_*	Brightness
**1**	–NMe_2_	DCM	500	727	2.04	6245	0.00042	8.63
		THF	499	690	2.18	5547	0.00084	18.36
		AcOEt	496	673	2.65	5302	0.00074	19.61
		Acetone	497	717	2.77	6174	0.00044	12.41
		MeOH	495	723	2.85	6371	0.00013	3.63
		NMP	505	710	2.24	5717	0.00071	15.95
		MeCN	495	732	2.82	6541	0.00042	1.18
		DMSO	508	716	1.75	5718	0.00042	7.04
**2**	–OEt	DCM	419	611	1.19	7500	0.00015	1.75
		THF	418	609	0.76	7503	0.00020	1.56
		AcOEt	417	606	1.48	7479	0.00013	1.93
		Acetone	417	605	1.63	7452	0.00013	2.12
		MeOH	415	609	1.31	7676	0.00015	1.79
		NMP	421	585	1.15	6659	0.00393	45.23
		MeCN	417	608	1.62	7533	0.00011	1.82
		DMSO	421	617	0.93	7545	0.00113	10.45
**3**	–*i*Pr	DCM	484	596	1.29	3882	0.00029	0
		THF	460	596	0.78	4961	0.00048	3.77
		AcOEt	461	588	1.14	4685	0.00013	1.49
		Acetone	460	591	1.14	4819	0.00008	0.91
		MeOH	485	576	1.17	3257	0	0
		NMP	471	555	1.04	3213	0.00033	3.44
		MeCN	475	586	1.43	3988	0	0
		DMSO	481	585	0.86	3696	0.00011	0.92
**4**	–H	DCM	479	592	1.43	3985	0	0
		THF	473	589	0.79	4164	0.00010	0.80
		AcOEt	472	595	1.15	4380	0.00011	1.22
		Acetone	472	593	1.13	4323	0.00007	0.83
		MeOH	478	583	1.39	3768	0.00019	2.69
		NMP	477	578	1.14	3663	0.00036	4.13
		MeCN	474	591	1.32	4176	0	0
		DMSO	480	591	1.09	3913	0.00086	0.94

DCM—dichloromethane, THF—tetrahydrofuran, AcOEt—ethyl acetate, MeOH—methanol, NMP—*N*-methyl-2-pyrrolidone, MeCN—acetonitrile, DMSO—dimethyl sulfoxide.

**Table 2:** Photophysical properties of compounds **5–8**.

No.	Substituent	Solvent	*λ_ab_* (nm)	*λ_fl_* (nm)	*ɛ* (× 10^4^, M^−1.^cm^−1^)	Stokes Shift (cm^−1^)	*ϕ*_fl_	Brightness
**5**	–NMe_2_	DCM	591	699	2.31	2614	0.13341	3083.93
		THF	585	687	2.08	2538	0.17281	3596.33
		AcOEt	580	685	2.28	2643	0.10533	2789.68
		Acetone	590	699	2.54	2643	0.00606	153.84
		MeOH	495	690	2.53	5709	0.02408	686.67
		NMP	608	711	1.78	2383	0.29677	5274.57
		MeCN	591	760	3.13	3762	0.00426	133.43
		DMSO	610	751	1.40	3078	0.77800	10810.62
**6**	–OEt	DCM	502	617	2.36	3713	0.02851	673.36
		THF	499	610	1.55	3647	0.02062	319.13
		AcOEt	486	606	1.45	4074	0.01394	202.67
		Acetone	488	611	1.64	4125	0.20587	338.84
		MeOH	458	618	1.38	5653	0	0
		NMP	471	624	1.30	5206	0.00046	6.00
		MeCN	496	618	2.13	3980	0.02167	460.87
		DMSO	469	617	1.13	5114	0.03301	430.85
**7**	–*i*Pr	DCM	487	591	1.50	3613	0.00972	145.83
		THF	483	585	1.76	3609	0.00626	110.09
		AcOEt	479	586	1.47	3811	0.00557	81.89
		Acetone	483	588	1.64	3697	0.00637	93.65
		MeOH	485	601	1.11	3979	0	0
		NMP	482	598	1.29	4029	0.00079	10.24
		MeCN	483	589	1.54	3726	0.00686	105.64
		DMSO	487	585	1.11	3440	0.01041	115.79
**8**	–H	DCM	482	579	1.69	3476	0.00527	89.41
		THF	478	578	1.17	3619	0.00355	41.27
		AcOEt	474	576	1.48	3736	0.00277	40.91
		Acetone	476	577	1.37	3677	0.00360	40.61
		MeOH	478	550	1.45	2739	0	0
		NMP	479	588	1.17	3870	0.00154	18.08
		MeCN	476	579	1.67	3737	0.00383	63.85
		DMSO	480	584	0.61	3710	0.00472	28.75

DCM—dichloromethane, THF—tetrahydrofuran, AcOEt—ethyl acetate, MeOH—methanol, NMP—*N*-methyl-2-pyrrolidone, MeCN—acetonitrile, DMSO—dimethyl sulfoxide.

**Table 3:** Energies of azo and hydrazone forms of 1-phenylazonaphthalen-2-ols (**1–4**) estimated in DMSO at the B3LYP/6-311++G(d,p) level of theory. ∆E represents the difference between azo and hydrazone form (∆E = E_a_−E_h_).

No.	Substituent	E (Hartree)		∆E (Hartree)	∆E (kcal/mol)
		Azo	Hydrazone		
**1**	–NMe_2_	−935.8620002	−935.8627862	0.0007860	0.493
**2**	–OEt	−955.7413224	−955.7435404	0.0022180	1.392
**3**	–*i*Pr	−919.8277747	−919.8312485	0.0034738	2.180
**4**	–H	−801.8518881	−801.8558497	0.0039616	2.486

**Table 4:** The values of hardness (η), energy gap, and energies of HOMO and LUMO orbitals estimated for all compounds **1–8**.

No.	Solvent	HOMO (eV)	LUMO (eV)	Energy Gap (eV)	η (eV)
**1** ^a^	DMSO MeOH DCM	−5.364 *−5.240* −5.362 *−5.238* −5.343 *−5.221*	−2.596 *−2.795* −2.591 *−2.790* −2.551 −*2.748*	2.768 *2.445* 2.771 *2.448* 2.792 *2.473*	1.384 *1.222* 1.385 *1.224* 1.396 *1.236*
**2** ^a^	DMSO MeOH DCM	−5.832 *−5.731* −5.829 *−5.728* −5.804 *−5.705*	−2.767 *−2.916* −2.763 *−2.912* −2.734 *−2.882*	3.066 *2.815* 3.066 *2.816* 3.070 *2.823*	1.533 *1.407* 1.533 *1.408* 1.535 *1.411*
**3** ^a^	DMSO MeOH DCM	−5.983 *−5.903* −5.980 *−5.900* −5.955 *−5.878*	−2.842 *−2.962* −2.839 *−2.959* −2.812 *−2.932*	3.141 *2.941* 3.142 *2.942* 3.143 *2.946*	1.571 *1.470* 1.571 *1.471* 1.572 *1.473*
**4** ^a^	DMSO MeOH DCM	−6.059 *−6.000* −6.057 *−5.998* −6.035 *−5.979*	−2.897 *−3.002* −2.894 *−2.999* −2.872 *−2.976*	3.162 *2.999* 3.162 *2.999* 3.163 *3.002*	1.581 *1.499* 1.581 *1.499* 1.582 *1.501*
**5**	DMSO MeOH DCM	−5.581 −5.580 −5.572	−3.245 −3.241 −3.206	2.336 2.339 2.366	1.168 1.170 1.183
**6**	DMSO MeOH DCM	−6.186 −6.185 −6.171	−3.404 −3.401 −3.378	2.782 2.783 2.793	1.391 1.392 1.396
**7**	DMSO MeOH DCM	−6.404 −6.403 −6.390	−3.459 −3.456 −3.436	2.946 2.946 2.953	1.473 1.473 1.477
**8**	DMSO MeOH DCM	−6.502 −6.501 −6.494	−3.500 −3.498 −3.483	3.002 3.003 3.010	1.501 1.502 1.505

^a^ for compounds **1–4** the values of both tautomeric forms were presented (azo form in standard font, hydrazone form in italic).

## Data Availability

Data sharing is not applicable for this article.

## Supplementary Materials

The following are available online at https://www.mdpi.com/article/10.3390/ma14123387/s1, Figure S1: ^1^H NMR spectrum (400 MHz) of 1-(4-dimethylamino)phenylazonaphthalen-2-ol (**1**) in CDCl_3_, Figure S2: ^13^C NMR spectrum (400 MHz) of 1-(4-dimethylamino)phenylazonaphthalen-2-ol (**1**) in CDCl_3_, Figure S3: ^1^H NMR spectrum (400 MHz) of 1-(4-ethoxy)phenylazonaphthalen-2-ol (**2**) in CDCl_3_, Figure S4: ^13^C NMR spectrum (400 MHz) of 1-(4-ethoxy)phenylazonaphthalen-2-ol (**2**) in CDCl_3_, Figure S5: ^1^H NMR spectrum (400 MHz) of 1-(4-isopropylo)phenylazonaphthalen-2-ol (**3**) in CDCl_3_, Figure S6: ^13^C NMR spectrum (400 MHz) of 1-(4-isopropylo)phenylazonaphthalen-2-ol (**3**) in CDCl_3_, Figure S7: ^1^H NMR spectrum (400 MHz) of 1-phenylazonaphthalen-2-ol (**4**) in CDCl_3_, Figure S8: ^13^C NMR spectrum (400 MHz) of 1-phenylazonaphthalen-2-ol (**4**) in CDCl_3_, Figure S9: ^1^H NMR spectrum (400 MHz) of 1-(4-dimethylamino)phenylazonaphthalen-2-ole difluoroborane (**5**) in CDCl_3_, Figure S10: ^13^C NMR spectrum (400 MHz) of 1-(4-dimethylamino)phenylazonaphthalen-2-ole difluoroborane (**5**) in CDCl_3_, Figure S11: ^1^H NMR spectrum (400 MHz) of 1-(4-ethoxy)phenylazonaphthalen-2-ole difluoroborane (**6**) in CDCl_3_, Figure S12: ^13^C NMR spectrum (400 MHz) of 1-(4-ethoxy)phenylazonaphthalen-2-ole difluoroborane (**6**) in CDCl_3_, Figure S13: ^1^H NMR spectrum (400 MHz) of 1-(4-isopropylo)phenylazonaphthalen-2-ole difluoroborane (**7**) in CDCl_3_, Figure S14: ^13^C NMR spectrum (400 MHz) of 1-(4-isopropylo)phenylazonaphthalen-2-ole difluoroborane (**7**) in CDCl_3_, Figure S15: ^1^H NMR spectrum (400 MHz) of 1-phenylazonaphthalen-2-ole difluoroborane (**8**) in CDCl_3_, Figure S16: ^13^C NMR spectrum (400 MHz) of 1-phenylazonaphthalen-2-ole difluoroborane (**8**) in CDCl_3_, Figure S17: Lippert-Mataga plot of compounds **1–8**, Figure S18: Bakhshiev plot of compounds **1–8**, Figure S19**:** Lippert-Mataga plots of compounds **1–4** (**a**) and **5–8** (**b**), Figure S20: Bakhshiev plots of compounds **1–4** (**a**) and **5–8** (**b**).
