# Supplementary material for: Substituent and Solvent Polarity on the Spectroscopic Properties in Azo Derivatives of 2-Hydroxynaphthalene and Their Difluoroboranes Complexes
Source: Materials (Basel). 2021 Jun 18;14(12):3387. doi: 10.3390/ma14123387 (PMC8235415; doi:10.3390/ma14123387)
Supplement: Supplementary file 1 [file materials-14-03387-s001.zip › materials-1258261-supplementary.pdf]

*Supplementary materials*

# Substituent and Solvent Polarity on the Spectroscopic Properties in Azo Derivatives of 2-Hydroxynaphthalene and Their Difluoroboranes Complexes

**Agnieszka Skotnicka [1, *] and Przemysław Czeleń [2]**

[1] Faculty of Chemical Technology and Engineering, UTP University of Science and Technology, Seminaryjna 3, 85-326 Bydgoszcz, Poland

[2] Department of Physical Chemistry, Faculty of Pharmacy, Collegium Medicum, N. Copernicus University, Kurpińskiego 5, 85-950 Bydgoszcz, Poland; przemekcz@cm.umk.pl

* Correspondence: askot@utp.edu.pl; Tel.: +48-(52)-3749-111

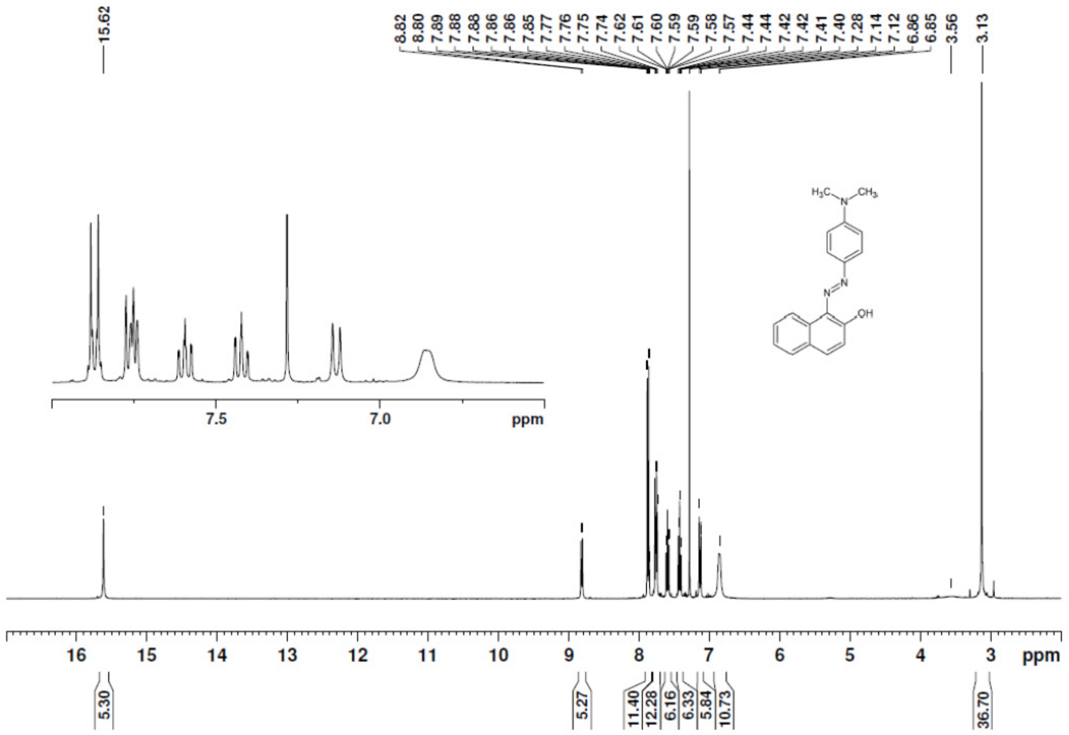

**Figure S1.** $^1$H NMR spectrum (400 MHz) of 1-(4-dimethylamino) phenylazonaphthalen-2-ol (**1**) in $CDCl_3$.

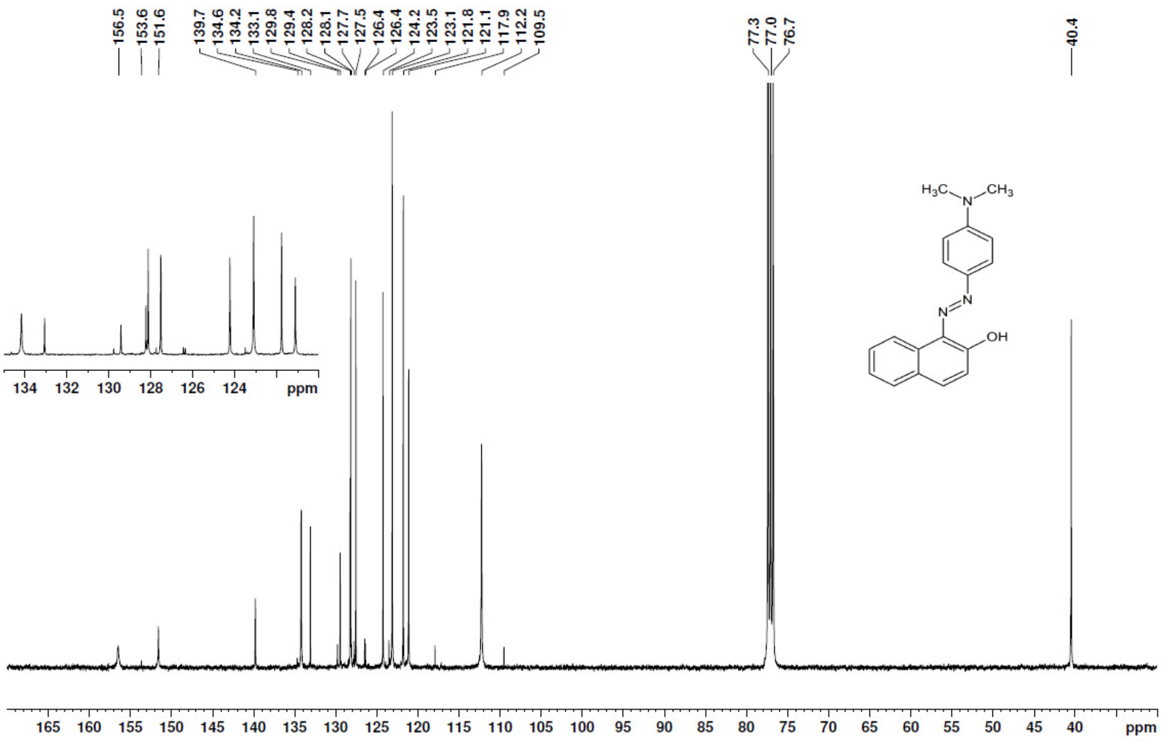


**Figure S2.** $^{13}C$ NMR spectrum (400 MHz) of 1-(4-dimethylamino)phenylazonaphthalen-2-ol (**1**) in $CDCl_3$.

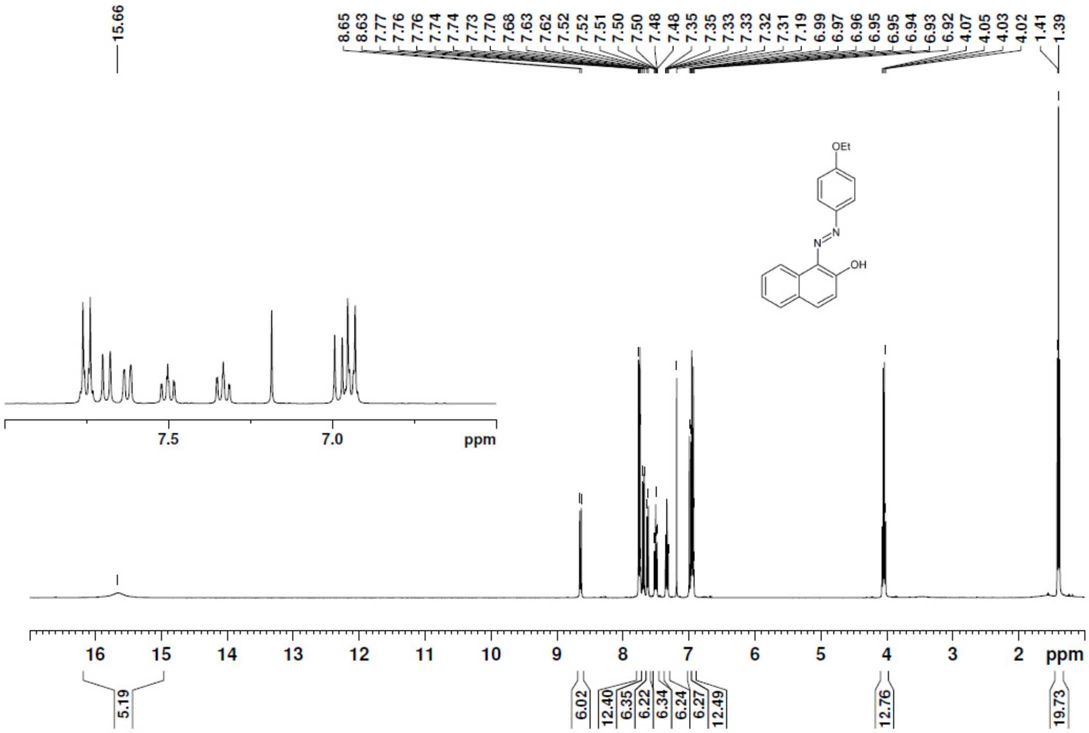


**Figure S3.** $^{1}H$ NMR spectrum (400 MHz) of 1-(4-ethoxy)phenylazonaphthalen-2-ol (**2**) in $CDCl_3$.

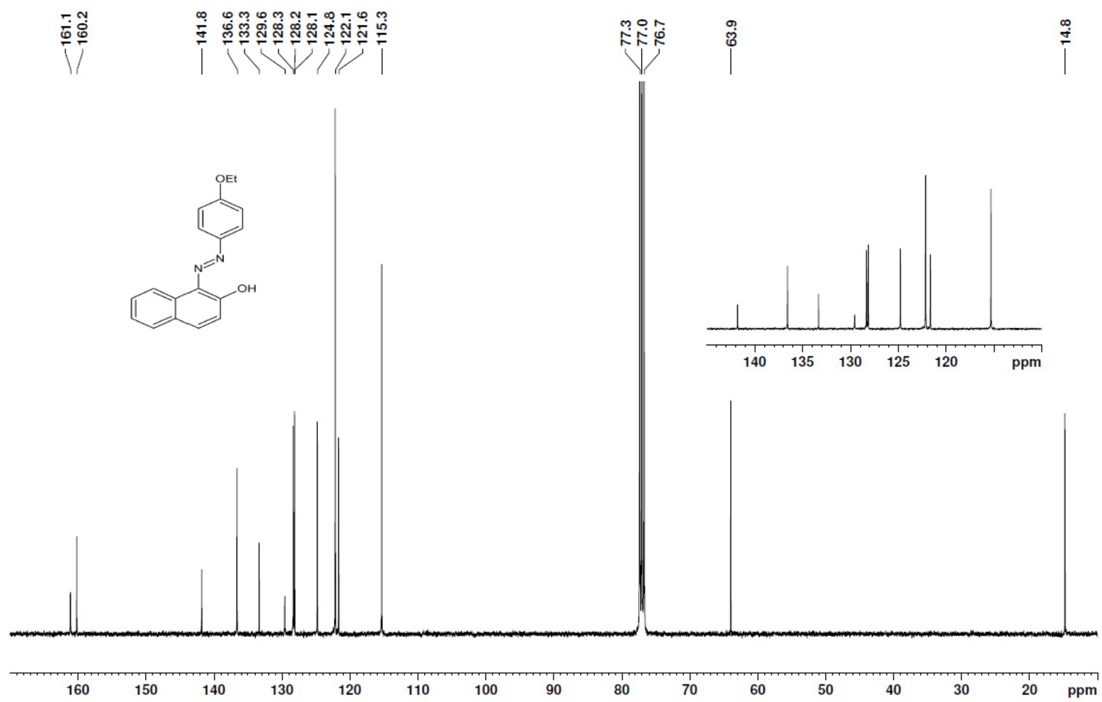


**Figure S4.** $^{13}C$ NMR spectrum (400 MHz) of 1-(4-ethoxy)phenylazonaphthalen-2-ol (**2**) in $CDCl_3$.

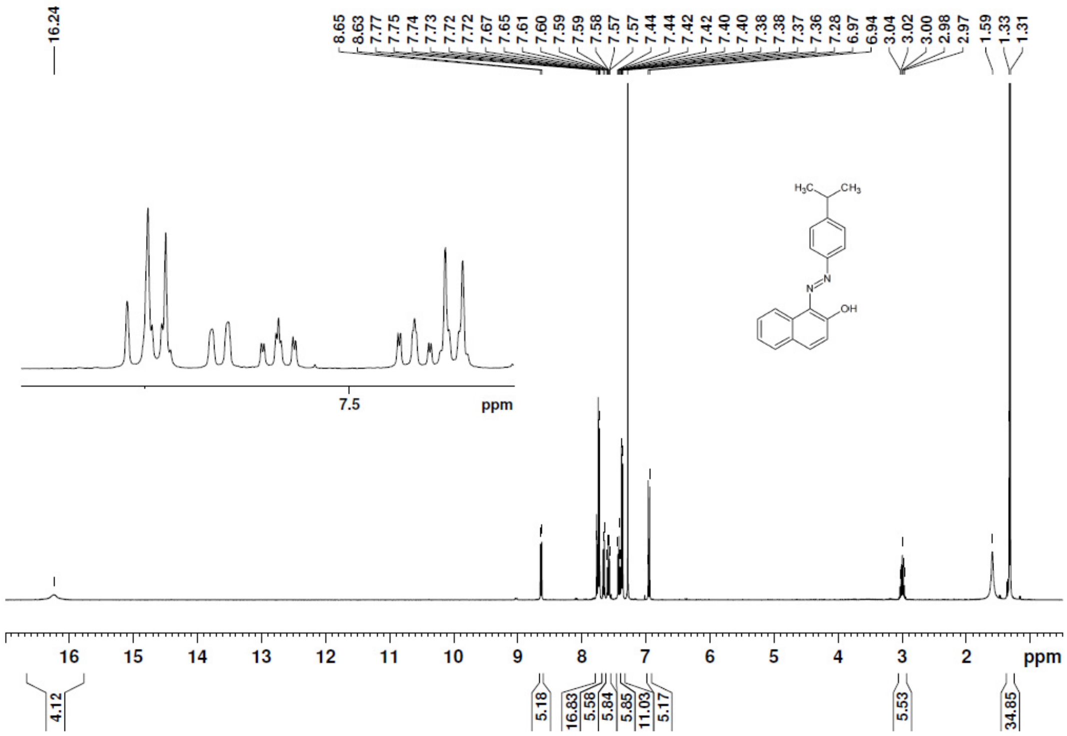


**Figure S5.** $^{1}H$ NMR spectrum (400 MHz) of 1-(4-isopropylo)phenylazonaphthalen-2-ol (**3**) in $CDCl_3$.

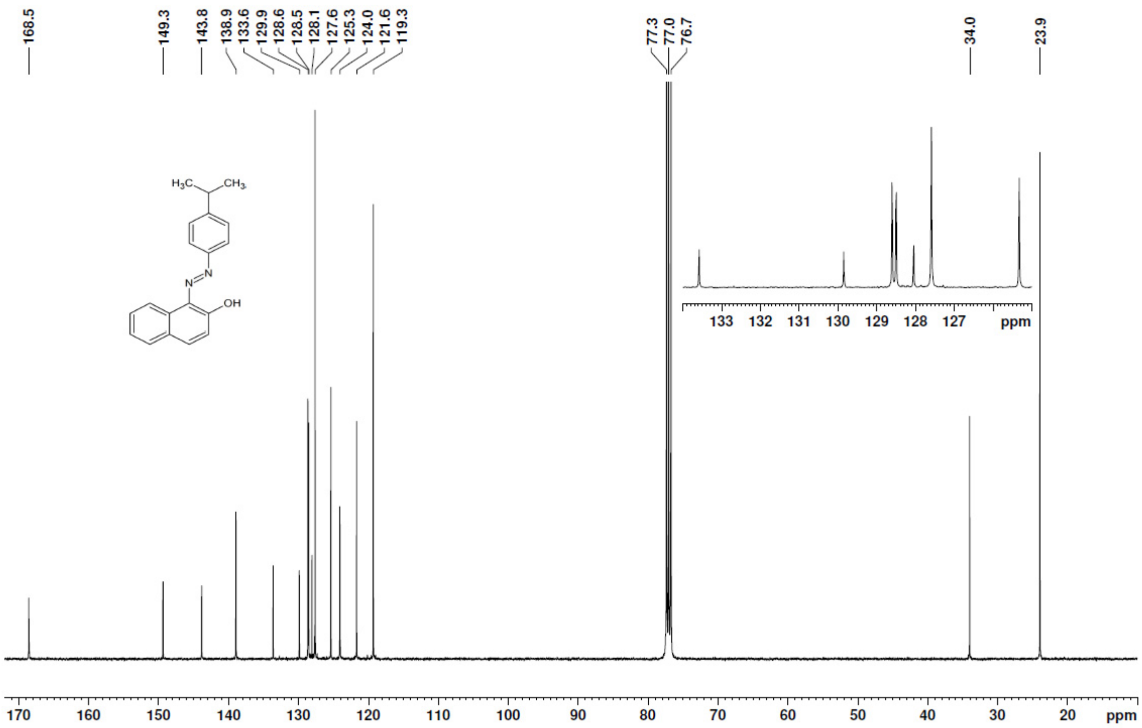


**Figure S6.** $^{13}C$ NMR spectrum (400 MHz) of 1-(4-isopropylo)phenylazonaphthalen-2-ol (**3**) in $CDCl_3$.

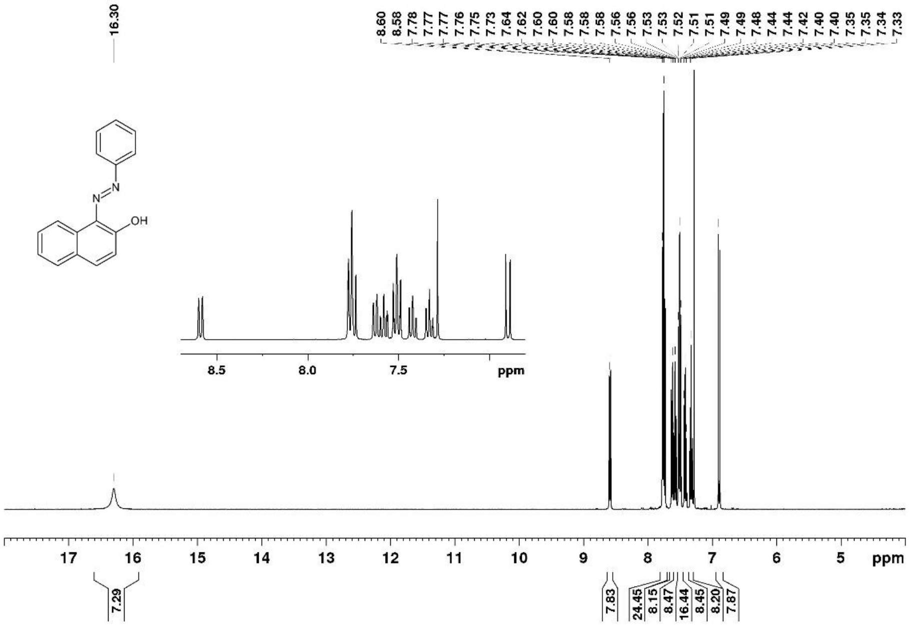


**Figure S7.** $^1H$ NMR spectrum (400 MHz) of 1-phenylazonaphthalen-2-ol (**4**) in $CDCl_3$.

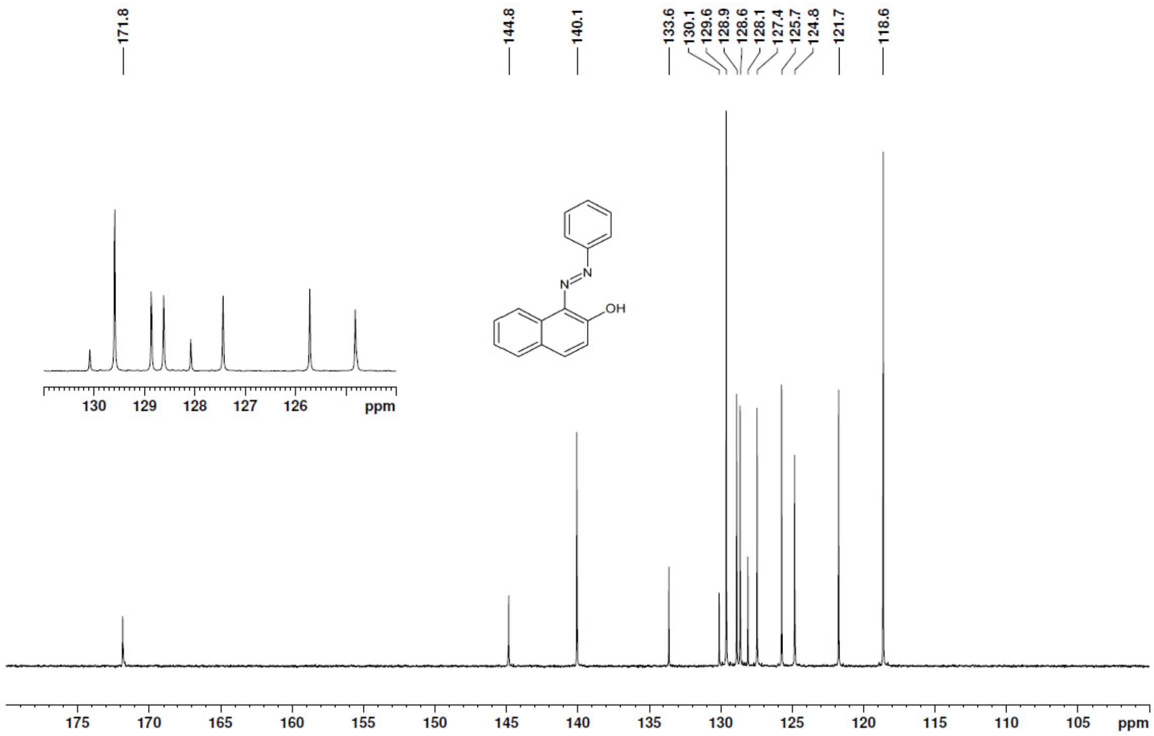


**Figure S8.** $^{13}C$ NMR spectrum (400 MHz) of 1-phenylazonaphthalen-2-ol (**4**) in $CDCl_3$.

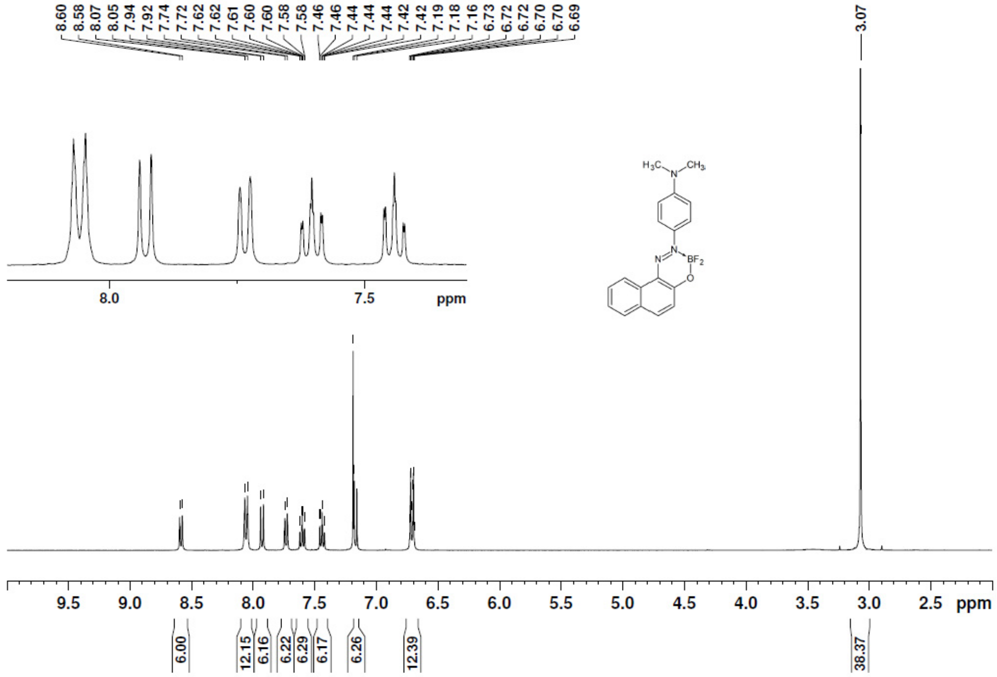


**Figure S9.** $^{1}H$ NMR spectrum (400 MHz) of 1-(4-dimethylamino)phenylazonaphthalen-2-ole difluoroborane (**5**) in $CDCl_3$.

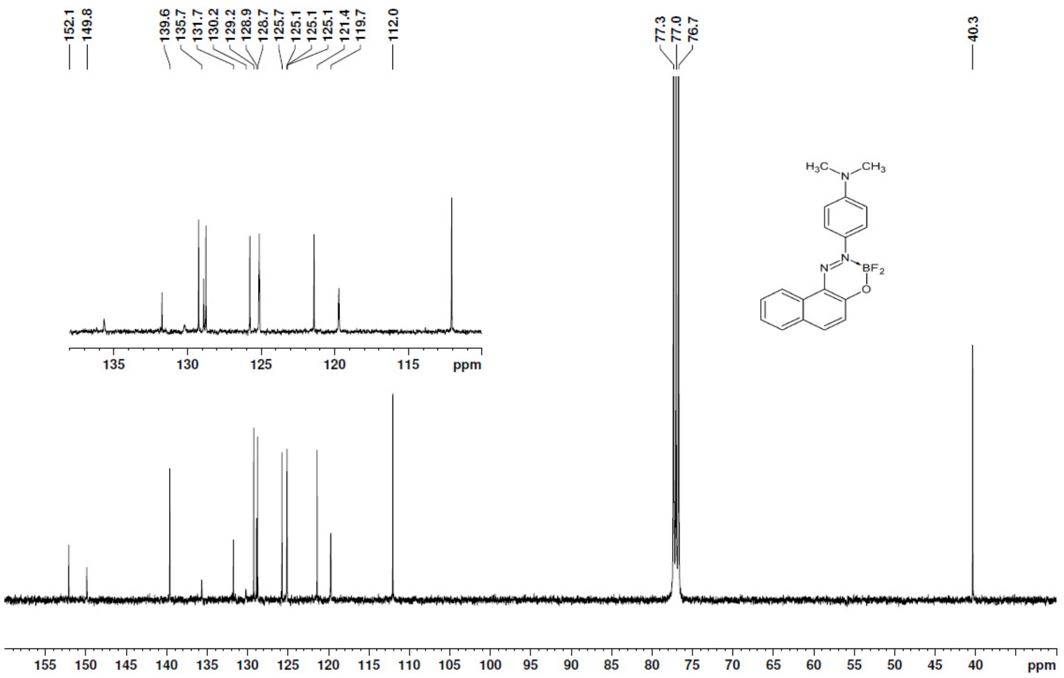


**Figure S10.** $^{13}C$ NMR spectrum (400 MHz) of 1-(4-dimethylamino)phenylazonaphthalen-2-ole difluoroborane (**5**) in $CDCl_3$.

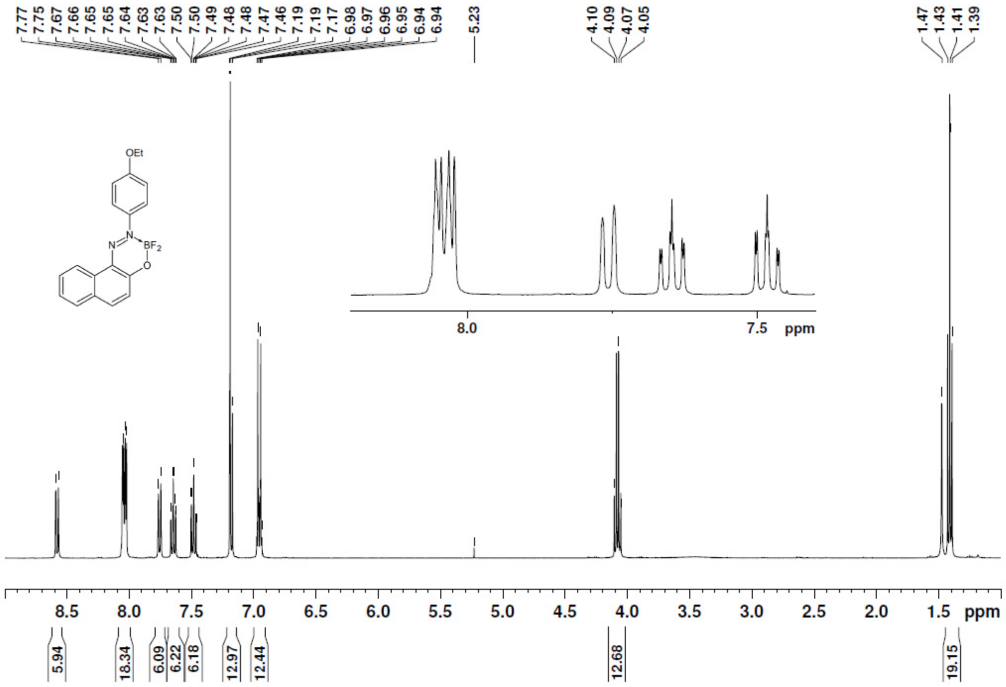


**Figure S11.** $^{1}H$ NMR spectrum (400 MHz) of 1-(4-ethoxy)phenylazonaphthalen-2-ole difluoroborane (**6**) in $CDCl_3$.

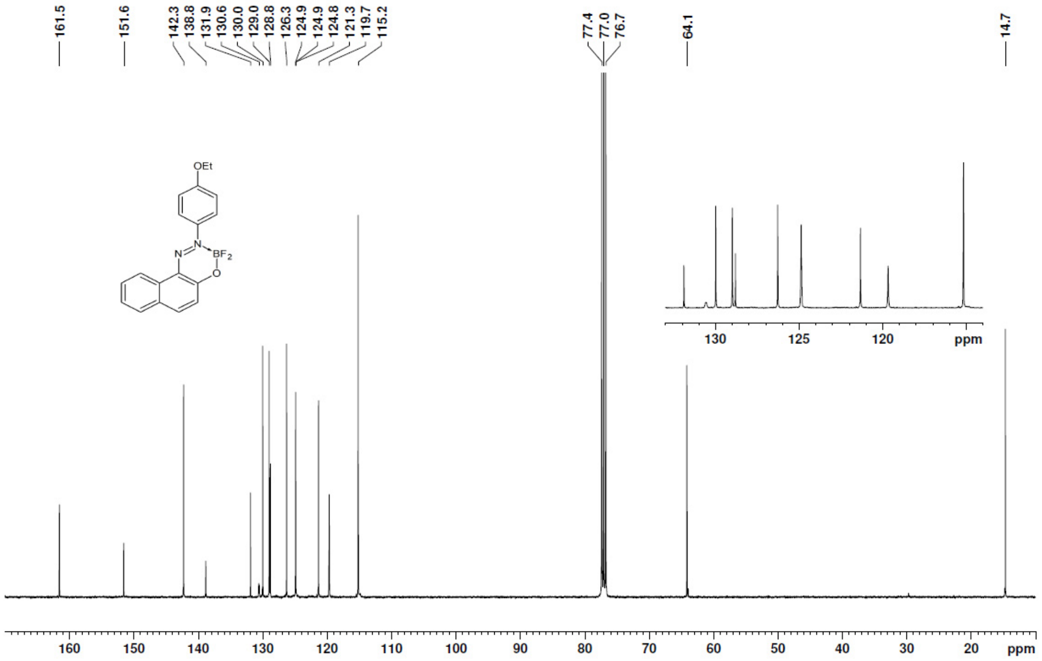


**Figure S12.** $^{13}$C NMR spectrum (400 MHz) of 1-(4-ethoxy)phenylazonaphthalen-2-ole difluoroborane (**6**) in $CDCl_3$

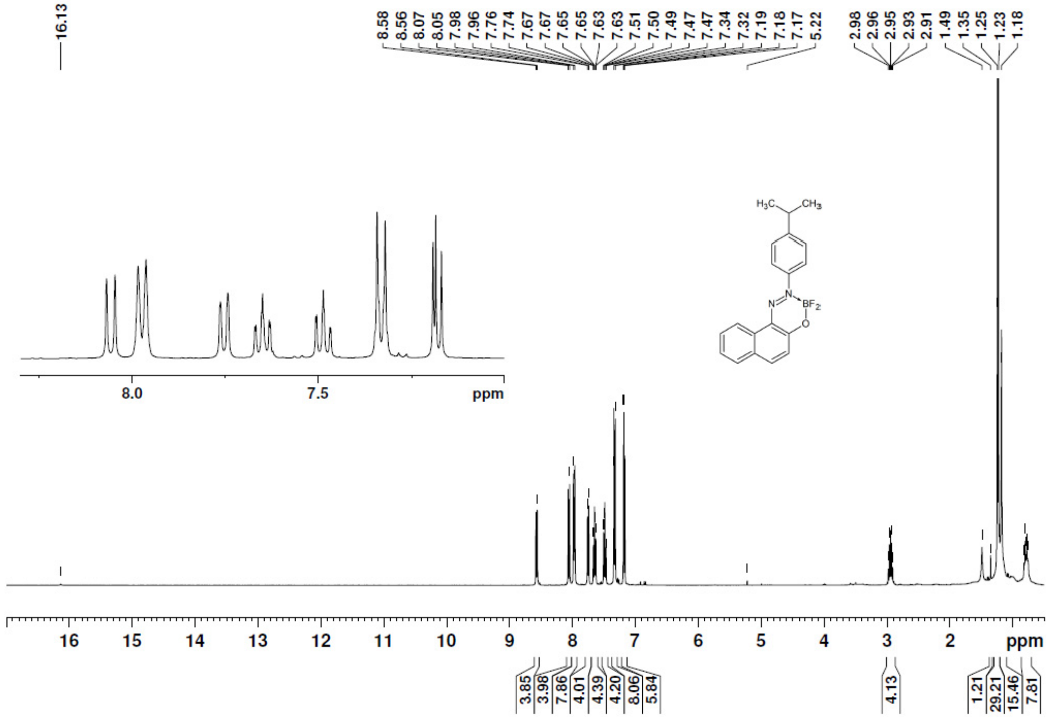


**Figure S13.** $^{1}$H NMR spectrum (400 MHz) of 1-(4-isopropylo)phenylazonaphthalen-2-ole difluoroborane (**7**) in $CDCl_3$.

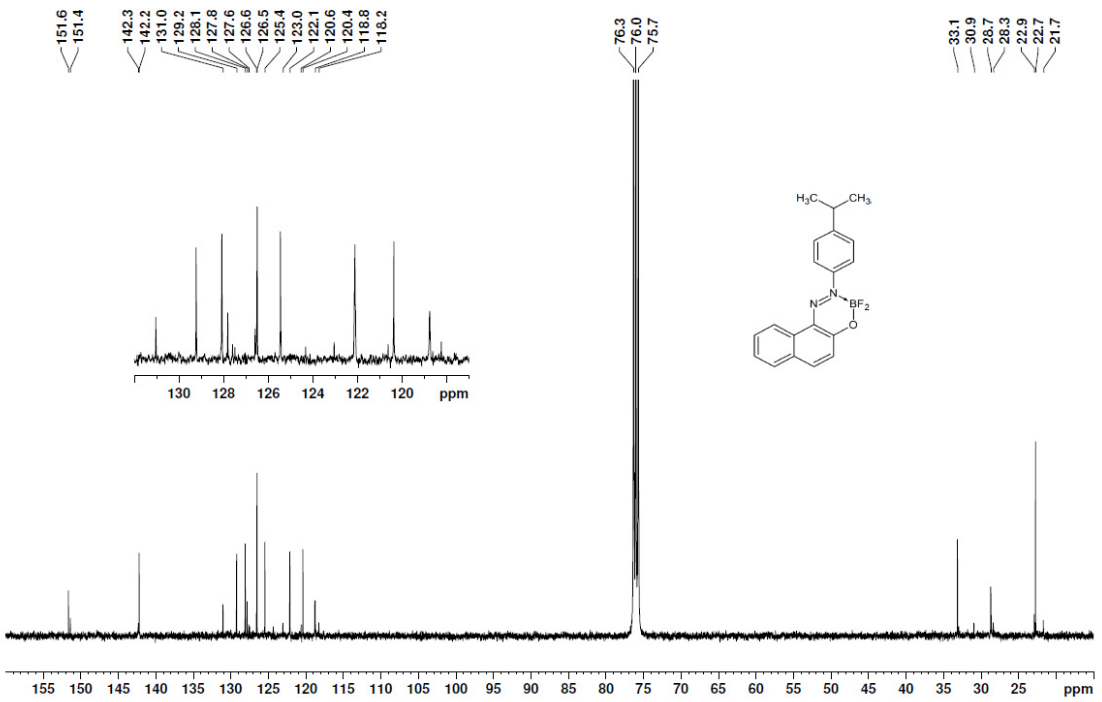


**Figure S14.** $^{13}C$ NMR spectrum (400 MHz) of 1-(4-isopropylo)phenylazonaphthalen-2-ole difluoroborane (**7**) in $CDCl_3$.

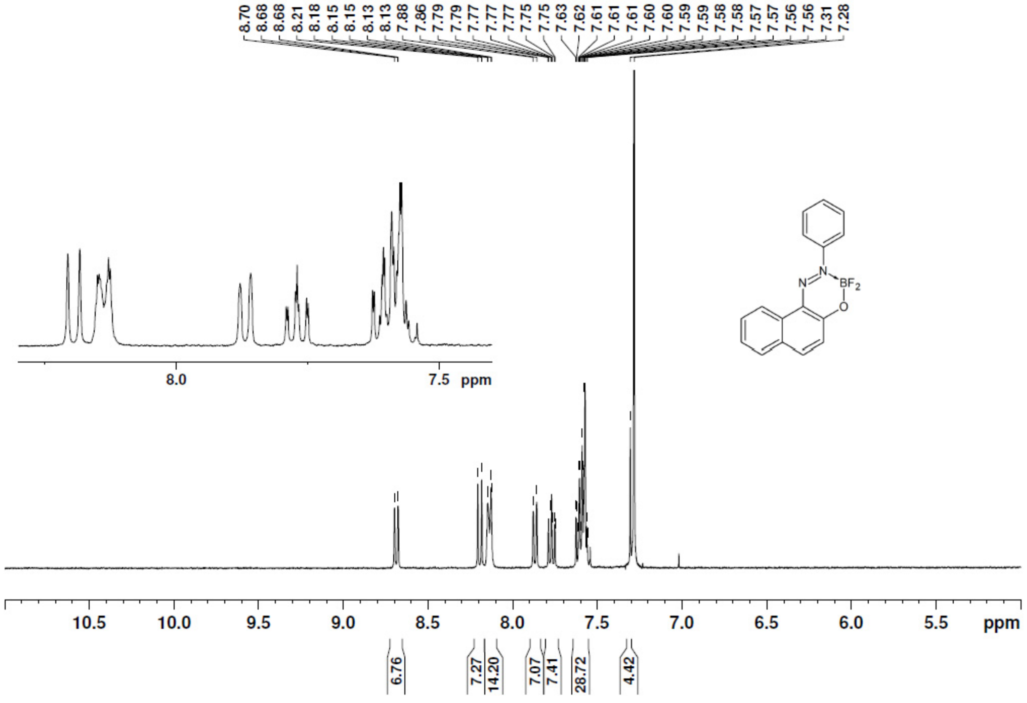


**Figure S15.** $^{1}H$ NMR spectrum (400 MHz) of 1-phenylazonaphthalen-2-ole difluoroborane (**8**) in $CDCl_3$.

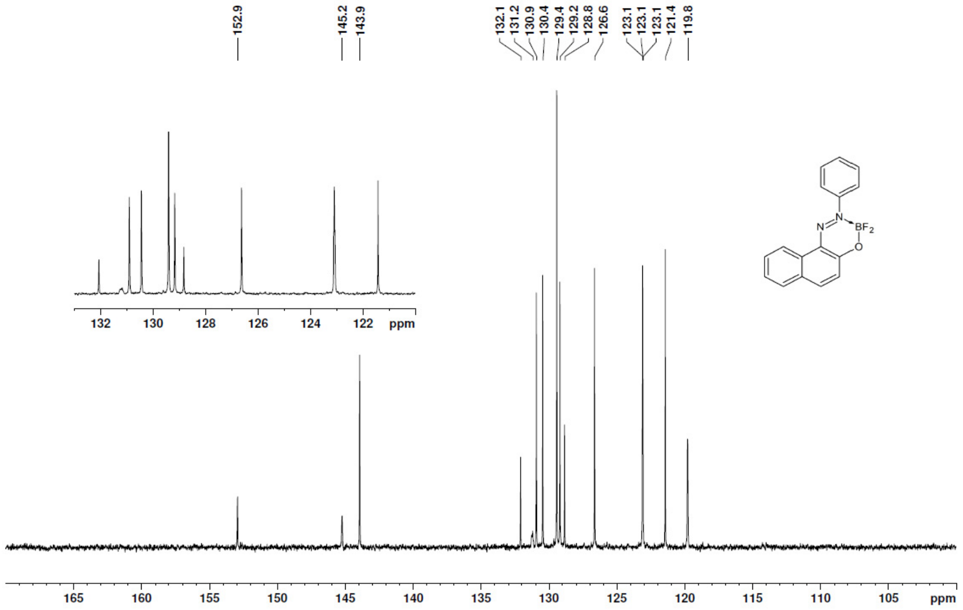


**Figure S16.** $^{13}$C NMR spectrum (400 MHz) of 1-phenylazonaphthalen-2-ole difluoroborane (**8**) in $CDCl_3$.

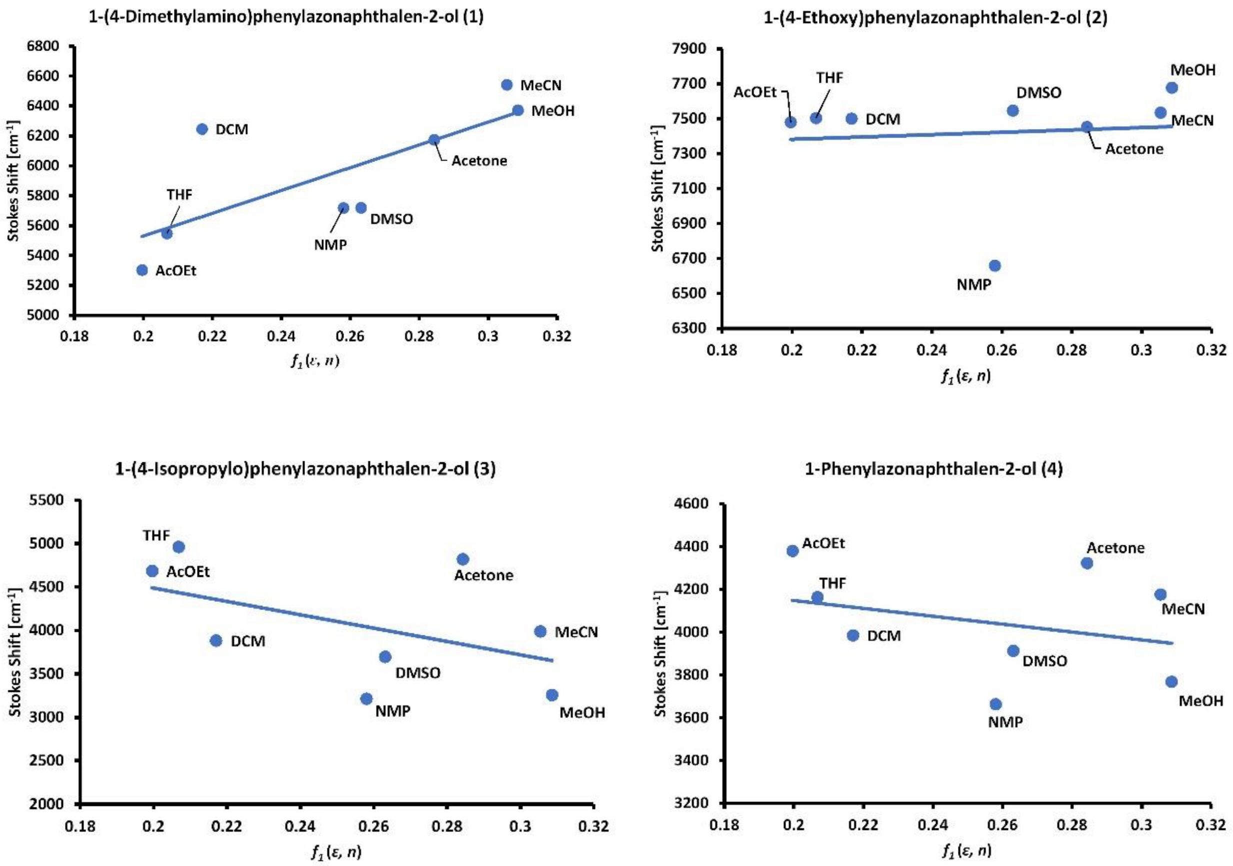

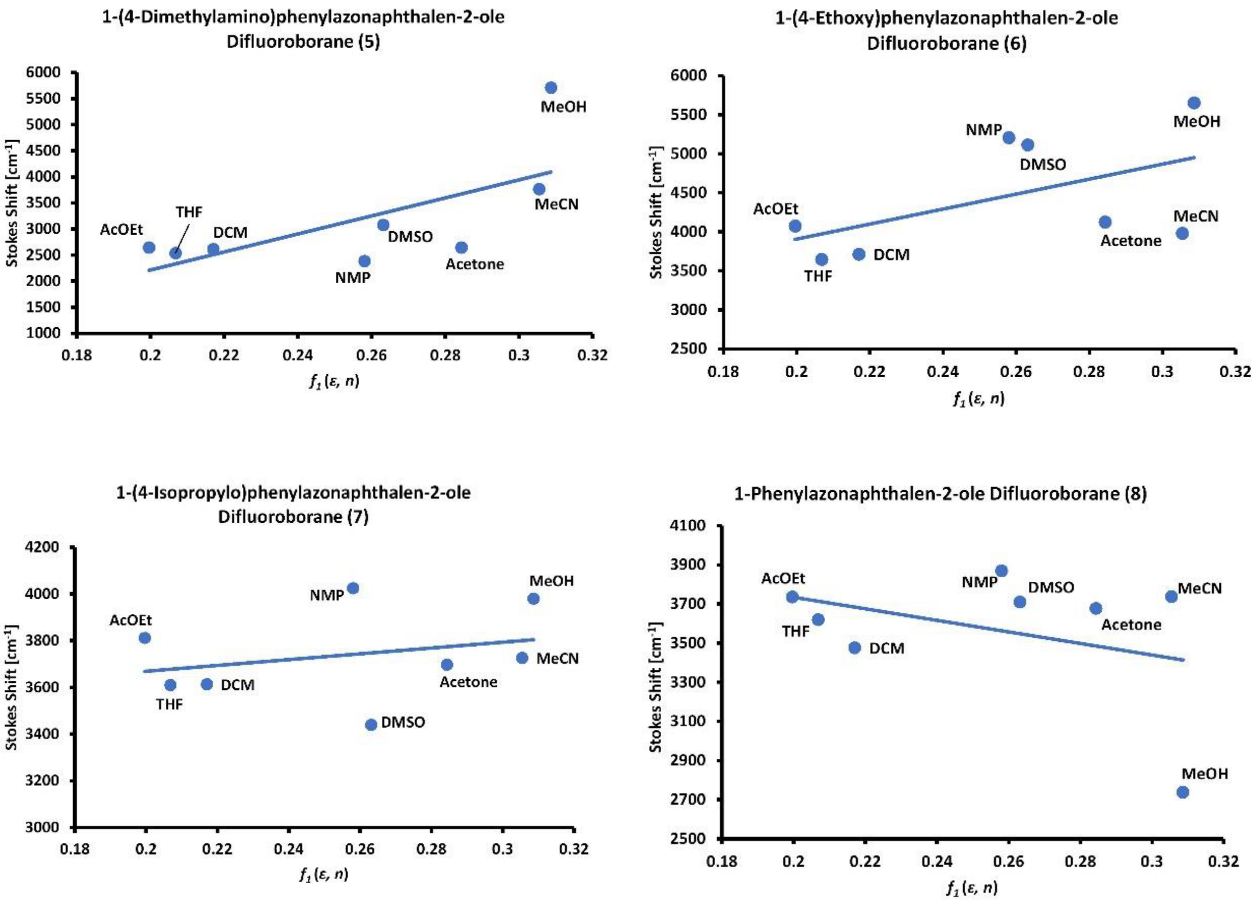


**Figure S17.** Lippert-Mataga plot of compounds **1**-**8**.

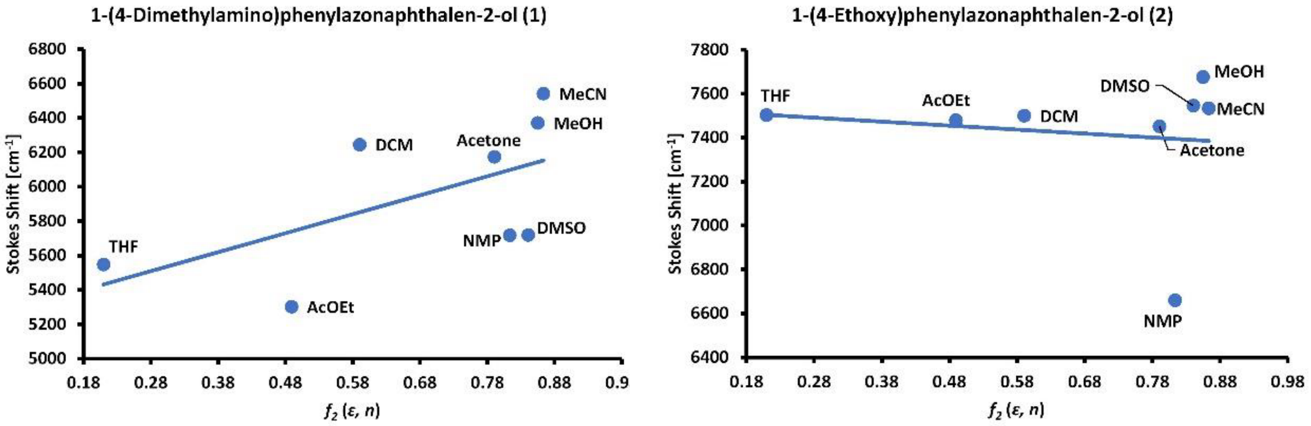

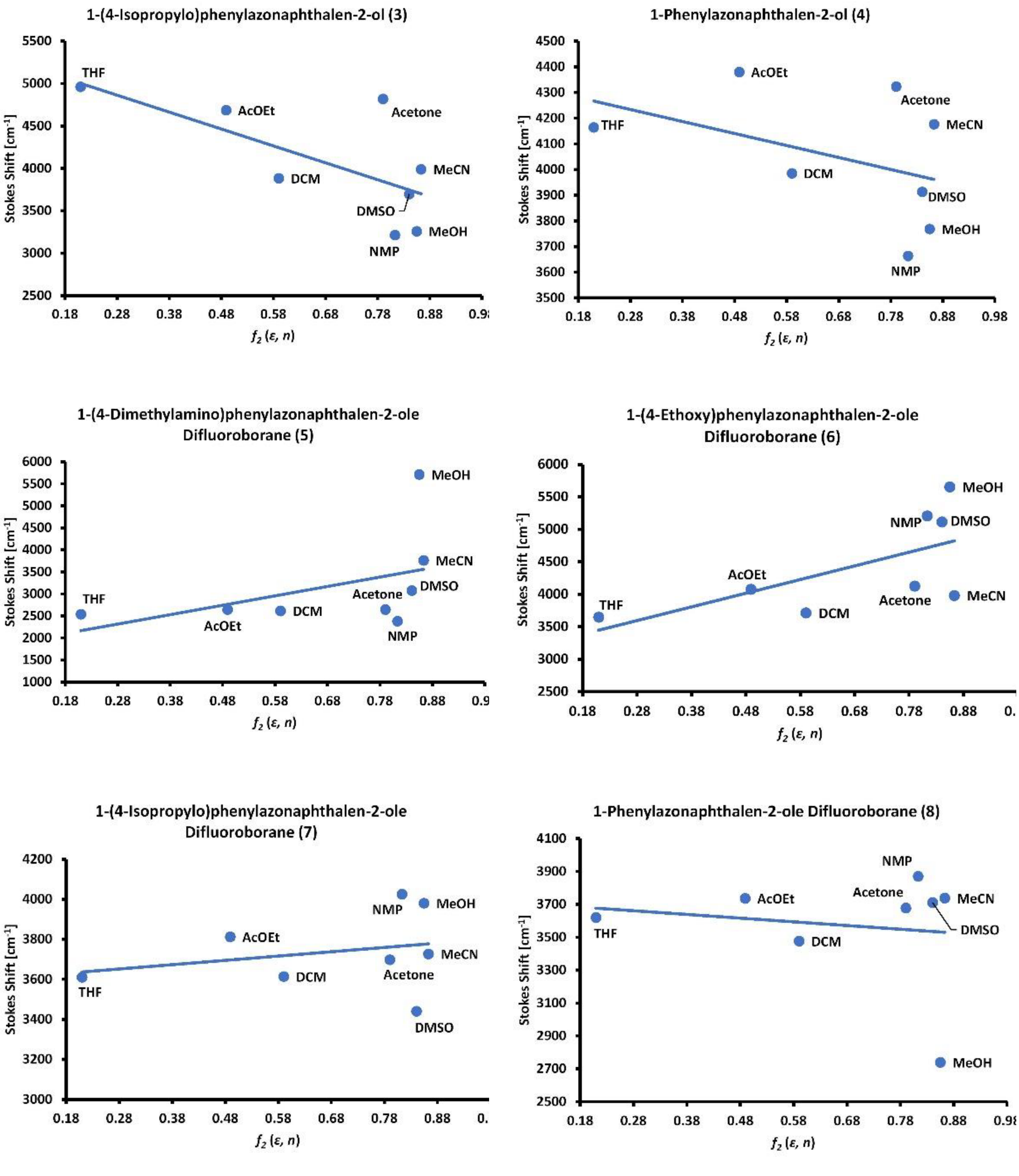


**Figure S18.** Bakhshiev plot of compounds **1**-**8**.

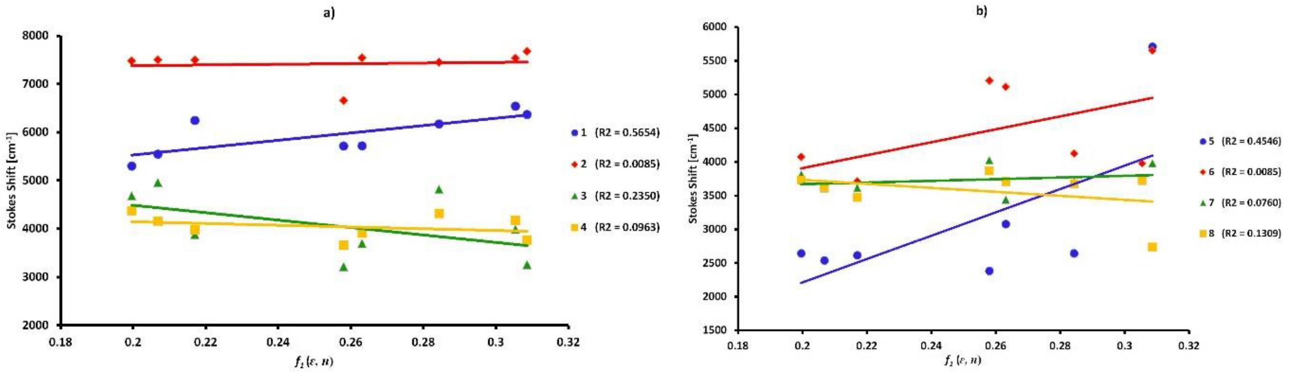


**Figure S19.** Lippert-Mataga plots of compounds **1**-**4** (**a**) and **5**-**8** (**b**).

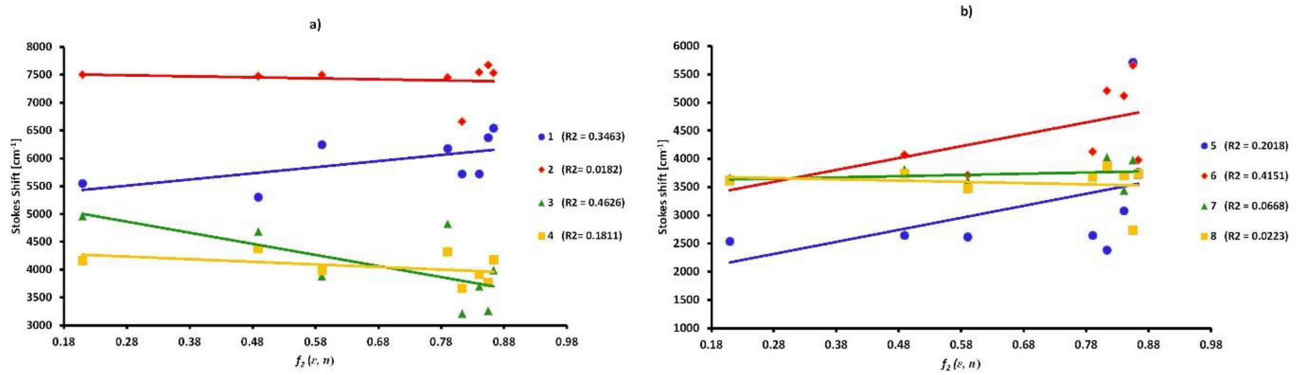


**Figure S20.** Bekhshiev plots of compounds **1**-**4** (**a**) and **5**-**8** (**b**).
